# Comprehensive Bio-Screening of Phytochemistry and Biological Capacity of *Oregano* (*Origanum vulgare*) and *Salvia triloba* Extracts against Oral Cariogenic and Food-Origin Pathogenic Bacteria

**DOI:** 10.3390/biom14060619

**Published:** 2024-05-24

**Authors:** Maria Antoniadou, Georgios Rozos, Natalia Vaou, Konstantinos Zaralis, Caglar Ersanli, Athanasios Alexopoulos, Aikaterini Dadamogia, Theodoros Varzakas, Athina Tzora, Chrysoula (Chrysa) Voidarou

**Affiliations:** 1 Department of Dentistry, School of Health Sciences, National and Kapodistrian University of Athens, 11527 Athens, Greece; 2 Executive Mastering Program in Systemic Management (CSAP), University of Piraeus, 18451 Piraeus, Greece; 3 Department of Agriculture, School of Agricultural Sciences, University of Western Macedonia, 53100 Florina, Greece; clevervet@hotmail.com (G.R.); kzaralis@uowm.gr (K.Z.); 4 Laboratory of Animal Health, Food Hygiene and Quality, Department of Agriculture, University of Ioannina, 47100 Arta, Greece; c.ersanli@uoi.gr (C.E.); kdadamogia@gmail.com (A.D.); tzora@uoi.gr (A.T.); 5 Laboratory of Microbiology, Department of Medicine, National and Kapodistrian University of Athens, 11527 Athens, Greece; nvaou@hotmail.com; 6 Laboratory of Microbiology, Biotechnology & Hygiene, Department of Agricultural Development, Democritus University of Thrace, 68200 Orestiada, Greece; alexopo@agro.duth.gr; 7 Department Food Science and Technology, University of the Peloponnese, 24100 Kalamata, Greece; t.varzakas@uop.gr

**Keywords:** *Origanum vulgare*, *Salvia triloba* extracts, phytochemistry and biological capacity, oral cariogenic and food-origin pathogenic bacteria

## Abstract

This study utilized phytochemical screening to conduct the qualitative analysis of plant extracts, aiming to identify various classes of secondary metabolites. Moreover, the antibacterial activity of different types of *Oregano vulgare* and *Salvia triloba* extracts was determined. To achieve the aim of this study, aqueous, ethanolic, and enzymatic extracts were prepared and screened for phytochemical capacity and antioxidant activities. The determination of the antibacterial activity included phenotypic screening of antibiotic susceptibility pattern of oral and food pathogenic bacterial strains, determination of the minimum inhibitory concentration and minimum bactericidal concentration—via microdilution broth test and in vitro valuation of antibacterial efficacies—of the anti-biofilm properties of the studied herbal extractions. Results: Our study evaluated the phytochemical composition and the antioxidant, antibacterial, and anti-biofilm properties of *O. vulgare* and *S. triloba* extracts. The analyzed samples contained bioactive compounds, such as phenolics and flavonoids, contributing to the observed strong antioxidant effect. Furthermore, they exhibited notable activity against oral biofilm formation and demonstrated significant antibacterial efficacy against dental caries’ microorganisms as well as food pathogens. Despite methodological variations, all extracts showed significant antioxidant capacity and promising antibacterial activity against various pathogens, including resistant strains, while also inhibiting biofilm formation. Although limited to two plant species and facing methodological constraints, this study lays the groundwork for future research, indicating the therapeutic potential of *O. vulgare* and *S. triloba* extracts. Further exploration is needed to report on underlying mechanisms and validate efficacy through clinical trials.

## 1. Introduction

Since ancient times, humans have recognized the capacity of plants to generate chemical substances essential for the survival of all organisms inhabiting planet Earth [1]. Both the cause of Socrates’ death and the development of active concentrations of salicylic acid (aspirin) involve these chemicals and they are among at least 100,000 chemical substances found in plants [2].

Phytochemistry divides plant metabolic products into two main categories: primary metabolites and secondary metabolites [3]. Although the conventional categorization of plant-derived organic compounds into primary metabolites, secondary metabolites, and hormones has been valuable over the years, recent studies have shed light on the complex interplay among different classes of plant secondary metabolites and plant metabolism [4]. Secondary metabolism accounts for 10% of a plant’s total metabolic processes and involves biosynthetic pathways that produce molecules not directly involved in essential cellular functions, such as growth and photosynthesis [5]. These pathways play a variety of ecological roles including the establishment of symbiotic relationships, pollination, interspecies competition, and chemically mediated plant–plant interference (i.e., allelopathy). Contrary to earlier beliefs that deemed secondary metabolites as the merely unnecessary by-products of primary metabolism, the current understanding recognizes them as critical extensions of primary metabolism [6]. These compounds fulfill numerous ecophysiological functions, such as defending plants against pathogens and herbivores, helping them manage abiotic stress, and aiding in reproduction and seed dispersal through mechanisms like pollinator attraction and allelopathy [7].

Secondary metabolites exhibit a wide range of diversity and are categorized into several major groups based on their biosynthetic pathways [8]. Phenolic compounds constitute one such group, characterized by the presence of at least one aromatic ring, synthesized through the shikimic and/or malonic acid pathways [4]. Terpenes and steroids, another category, are produced via the acetyl coenzyme A through the mevalonate pathway in the cytoplasm and the pyruvate-phosphoglyceraldehyde pathway in plastids [8]. Nitrogenous secondary metabolites primarily derive from amino acids, comprising the third group. Lastly, phytohormones, also recognized as plant hormones, play crucial roles in regulating various organismal processes and cellular activities within plants. These hormones, belonging to different chemical groups, including indole derivatives, sesquiterpenes, alkenes, diterpenoid acids, aliphatic nitrogenous bases, phenolic organic acids, and terpenoid lactones, orchestrate diverse biological functions and biosynthesis within plants [9]. Understanding the concepts outlined above naturally prompts the inquiry into how chemicals synthesized by one organism might impact or be harnessed by another belonging to other classes. This line of thought gives rise to the idea of utilizing plants in their entirety or extracting specific chemicals from them to create formulations with antimicrobial properties, as well as antioxidant attributes [10].

Moreover, the exploration of the oral microbiota is an ever evolving and captivating field of study [11,12]. Its importance transcends oral health alone, as numerous oral diseases, including dental caries and periodontitis, are linked to various non-communicable conditions such as diabetes, cardiovascular diseases, pneumonia, obesity, cancers, and premature birth [13,14]. Distributed within biofilms throughout the oral cavity, the oral microbiome forms a plethora of micro-ecosystems crucial for maintaining health equilibrium [15]. Any disruptions to this delicate balance can pave the way for the emergence of pathogens and subsequent disease [16]. Moreover, serving as the primary gateway to the gastrointestinal system, the oral cavity acts as the initial point of entry for xenobiotics into the body [17]. Imbalances in the diverse microecosystems within the oral cavity can lead to dysbiosis, highlighting the imperative of identifying the microbiome in a healthy state [18]. Therefore, there is an urgent need to investigate chemical substances derived from natural sources, as well as to design synthetic or semi-synthetic compounds that can act as regulators of biological processes such as inflammation and oxidative stress within the oral cavity. These compounds should also serve as effective antimicrobial agents against oral (especially cariogenic) pathogens.

Phytochemical research is expected to reveal undiscovered biomolecules from which pioneering remedies could develop [19,20,21]. This expectation becomes especially relevant as the threat of antibiotic resistance posed by pathogenic bacteria escalates significantly each year [22,23,24,25,26]. It is also true that the pharmaceutical industry faces serious economical, regulatory, and scientific difficulties to develop new classes of antibiotics [27]. Developing novel strategies to combat antibiotic resistance is now paramount [14,20]. Under these dire perspectives, plant extracts are recognized as a unique and valuable source of natural substances useful for drug discovery and advancement [20,28,29]. In the present research, we focus on the in vitro biological evaluation of the extracts of two plants: *Origanum vulgare (O. vulgare)* obtained from the mountainous regions of Epirus, Greece, and *Salvia triloba (S. triloba)*, commonly known as Greek sage, originating from the island of Crete, Greece. The aim of this study is to conduct a screening involving the qualitative analysis of these plant extracts to identify their antioxidant, antibacterial, and anti-inflammatory actions.

## 2. Materials and Methods

**Scheme 1:**
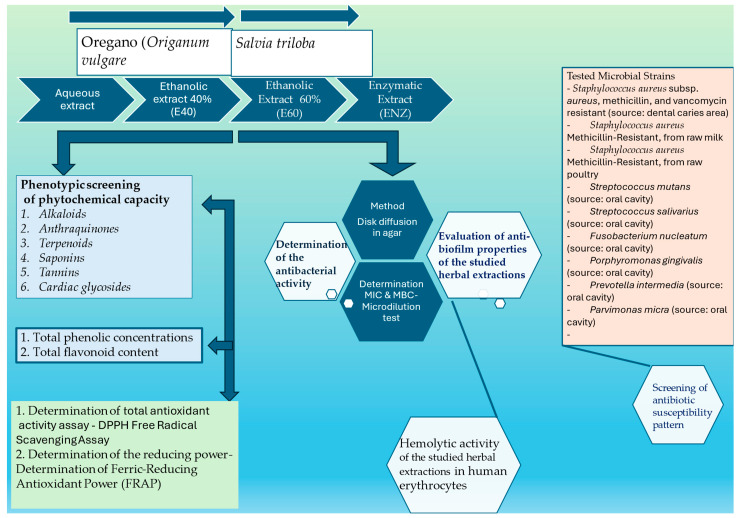
The schematic representation of the experimental flow setup.

The schematic representation of the experimental flow setup is depicted in Scheme 1. An individual text field for explanations is provided below, primarily focusing on the methods or parameters requiring further clarification.

### 2.1. Plant Materials

The present study examined two plant species: (a) Wild oregano plants (*Origanum vulgare*) were collected in July from a mountainous area (750 m in altitude) close to Stephaniada lake in the Epirus district in NW Greece (39°26′ N, 21°49′ E); and (b) Greek sage plants (*Salvia triloba*) were collected after flowering (in September) from a wild population in the district of Apokoronas in western Crete at an altitude of approx. 250 m (35°29′ Ν, 24°36′ E). Samples of these plants, gathered by locals and after scanning a voucher specimen of each plant species, were deposited in the herbarium of the Department of Agricultural Development at the Democritus University of Thrace with a reference number DAD-LM-76 (*Origanum vulgare*) and DAD-LM-79 (*Salvia triloba*). The studied plants were air-dried at room temperature and then divided into distinct parts, including flowers, roots, leaves, bark, and stems. After complete drying, the samples were ground into a fine powder using a high-speed grinder, focusing only on the leaves and flowers. The powdered plant materials were stored at −18 °C for further analysis.

#### 2.1.1. Preparation Methods for Plant Extracts

The maceration method was employed for the preparation of the aqueous, ethanolic, and enzymatic extracts [30].

#### 2.1.2. Aqueous Extract (A)

Each plant powder was mixed with distilled water (DIW) at a ratio of 1:2 (*w*/*v*) and suspended in sterile flasks with continuous stirring at 30 °C for 24 h. Afterward, the mixture was filtered through sterilized Whatman No. 1 filter paper, and the resulting liquid extracts were stored at −80 °C for 24 h. Concentration was achieved through evaporation under reduced pressure using a rotary evaporator (KNFRC 900; KNF Neuberger GmbH, Breisgau, Germany). Aqueous extracts underwent lyophilization in a freeze dryer for approximately 7 h or overnight. Prior to use, these extracts were exposed to ultraviolet light overnight to eliminate potential microbial contaminants [1]. The letter “A” was used as an abbreviation for convenience.

#### 2.1.3. Ethanolic Extract (E)

To obtain ethanol extracts, we followed the procedure outlined in Section 2.1.2, with the only difference being the use of an ethyl alcohol solution as the solvent instead of DIW. Two concentrations of 96% ethyl alcohol solution were used: 40% and 60%, both prepared from DIW [1]. The abbreviations used for the ethanolic extracts were E40 and E60, representing 40% and 60% concentrations, respectively.

#### 2.1.4. Enzymatic Extract (ENZ)

One kilogram of pretreated fresh plant material (leaves and flowers) was immersed in a solution consisting of 2 kg of acidified DIW adjusted to pH = 2 using concentrated hydrochloric acid and 1.0% pepsin (Merck KGaA, Darmstadt, Germany) [24]. After an incubation period at 37 °C for 48 h, hydrolysis was stopped by heating for 10 min. The resulting solution was divided into smaller 200 g batches, manually compressed with a sterile pestle, and filtered through sterilized Whatman No. 1 filter paper. Subsequently, the solvent from each batch was evaporated using a rotary evaporator (KNFRC 900; KNF Neuberger GmbH, Breisgau, Germany). The final step involved deep freezing at −80 °C followed by lyophilization [1,31].

Keynotes for the pretreatment process: The plant material underwent several steps, including washing under running water, manual removal of unsuitable elements, and cutting into 1 mm pieces using a slicer to increase the surface area. These pieces were then washed with phosphate-buffered saline (PBS) to eliminate intracellular vesicles released from damaged cells [1,32]. The abbreviation “ENZ” was used. Samples were prepared by weighing the crude extracts mentioned above and determining the volume of solvent [5% aqueous solution of dimethyl sulfoxide (DMSO)] needed to create a sample stock solution with a concentration of 10 mg/mL. Aqueous extracts were dissolved using sterile distilled water.

### 2.2. Screening the Phytochemical Capacity and Antioxidant Activities of Plant Extracts

#### 2.2.1. Phytochemical Identification

To evaluate the phytochemical composition of the plant extracts, we conducted the following tests.

Clarification: For qualitative phytochemical screening, we prepared two types of aqueous extract solutions. The first was dissolved in sterile boiled water, designated as “A”, while the second, an aqueous extract dissolved in a solvent composed of a 1:1 ratio of boiled water and 95% methanol, was labeled as “A*”. All subsequent experiments were conducted in triplicate.

- Detection of alkaloids [1,32,33]

The alkaloids in the plant extracts were identified, using (a) Mayer’s reagent (1.36 g of mercuric chloride and 5.00 g of potassium iodide dissolved in 100 mL water, a precipitate of alkaloid compounds); (b) Wagner’s reagent (2 g of iodine and 6 g of potassium iodide dissolved in 100 mL of distilled water a precipitate of alkaloids); and (c) Hager’s reagent (Picric acid test, where a few drops of picric acid added to 5 mL of a plant extract leads to the formation of a yellow precipitate of alkaloids), as per the previous reports.

- Detection of anthraquinones [1,32,33]

In summary, fifty milligrams of the extract were dissolved in 5 mL of distilled water and then filtered. Two milliliters of the resulting extract solution were transferred to a test tube containing 10 mL of benzene. The mixture was vigorously shaken for 10 min and then filtered. Finally, 5 mL of a 10% ammonia solution was added to the test tube and shaken vigorously for an additional 30 s. The presence of free anthraquinones was indicated by the development of a pink, red, or violet color, evaluated based on a positivity rating.

- Detection of terpenoids

To detect the presence of tri-terpenoids, the Salkowski test was conducted using chloroform and concentrated sulfuric acid (H_2_SO_4_) as the reagents. A quantity of 5 mL was mixed with 2 mL of chloroform and then 3 mL of concentrated sulfuric acid was added to form a layer. The test was ranked as positive when a red–brown coloration appeared in the interface.

- Detection of saponins (foam test) [32]

Saponins were identified by the formation of persistent foam at 25 °C. To achieve this, 500 milligrams of the extract were dissolved in 2 mL of distilled water. The resulting suspension was vigorously shaken for 15 min in a graduated cylinder. The presence of saponins was confirmed by the formation of a 2 cm layer of foam.

- Detection of tannins (ferric chloride test—Braymer’s test) [33]

To assess tannin content, a mixture was prepared from each plant extract. This involved dissolving 500 milligrams of the extract in 5 mL of ethanol, followed by sonication at 40 kHz for 5 min using a sonicator, and centrifugation at 190× *g* for 10 min. Subsequently, 1 mL of supernatant was collected. Then, 1 mL of 15% ferric chloride (FeCl_3_) was added, and the formation of a dark green or blue–black precipitate indicated the presence of tannin.

- Detection of cardiac glycosides (Keller–Kiliani test) [32,33]

A total of 50 milligrams of the extract were dissolved in distilled water and subsequently filtered. To 2 mL of the filtrate, 1.5 mL of glacial acetic acid, a single drop of 5% ferric chloride (FeCl_3_), and a drop of concentrated sulfuric acid (H_2_SO_4_) along the inner walls of the test tube were added. The transition to green–blue coloration at the upper layer and reddish brown at the interface between the two layers confirmed the presence of cardiac glycosides.

#### 2.2.2. Total Phenolic Concentrations [34]

The concentration of total phenolic compounds in the plant extracts was assessed using the Folin–Ciocalteu method, following the procedure outlined in our previous study. Folin–Ciocalteu reagent (120 µL) was mixed with 15 µL of each plant extract (final concentrations ranging from 4 to 40 µg/mL in methanol) in a 96-well plate. After 5 min, 120 µL of Na_2_CO_3_ solution (60 g/L) was added to each well and mixed. The plate was then incubated in darkness for 90 min before measurement. Absorbance was recorded at 725 nm using a microplate reader. The total phenolic content was determined from the standard curve of gallic acid in methanol, with a final concentration range of 2–40 µg/mL. Finally, the total concentration of phenolic compounds was expressed as gallic acid equivalents (GAEs) in milligrams per gram of dry weight of the sample.

#### 2.2.3. Estimation of Total Flavonoid Content

The total flavonoid content was determined using the aluminum chloride assay, with catechin serving as the standard for expressing total flavonoid content in milligrams of catechin equivalents (CEs) per gram of dry weight of the sample (mg CEs/g of DW) [34,35]. The total flavonoid content in the extracts was determined as follows: A volume of 0.50 mL of each plant extract was added to a 10 mL volumetric flask containing 4 mL of deionized water (DIW). To this flask, 0.3 mL of 5% NaNO_2_ was added. After 5 min of incubation, 0.3 mL of 10% AlCl_3_ was added. At the 6th minute, 2 mL of 1 M NaOH was added, and DIW was added to reach the mark. An orange–yellowish color developed. After 10 min of incubation, the absorbance was measured at 510 nm.

#### 2.2.4. Total Antioxidant Activity Assay [35] (DPPH Free-Radical Scavenging Assay)

The 2,2-diphenyl-1-picrylhydrazyl (DPPH) free-radical scavenging assay was employed to assess the in vitro antioxidant activities of plant extracts, as detailed in our previous report.

#### 2.2.5. The Reducing Power Assay

The evaluation of the reducing power of the tested plant extracts is based on the principle that molecules with the potential to undergo reduction react with potassium ferricyanide (Fe^3+^), generating potassium ferrocyanide (Fe^2+^). This resulting compound then reacts with ferric chloride, forming a ferric ferrous complex that exhibits an absorption peak at 700 nm. A greater absorbance of the reaction mixture signifies a higher reducing power [1,35].

Clarification: Each sample underwent triplicate testing for all the assays mentioned in Section 2.2.

### 2.3. Determination of the Antibacterial Activity

#### 2.3.1. Tested Bacterial Strains

In this study, we examined nine (9) bacterial strains isolated from lesions of the oral cavity and food sources. These strains display diverse characteristics and have varying requirements for growth, nutrient substrates, and optimal incubation conditions.

The strains of the pathogenic bacteria that were tested as cell-targets are mentioned in Table 1.

All the mentioned strains were identified and classified using standard laboratory procedures from the protocols followed by the Departments of Medicine and Dentistry at the National and Kapodistrian University of Athens. In our present study, we introduced bacteria with diverse metabolic growth processes and nutritional requirements. As a result, we employed a variety of nutrient media and incubation conditions, as detailed in our previous publication [1].

##### Phenotypic Screening of Antibiotic Susceptibility Pattern of Bacterial Strains

Antibiotic susceptibility tests were conducted for all tested bacteria using the Kirby–Bauer disc diffusion method, following the guidelines set out by The National Committee for Clinical Laboratory Standards (later renamed The Clinical Laboratory Standard Institute (CLSI)). According to the relevant clinical guidelines, the following antibiotics were included in the analysis of antimicrobial profiles [36,37,38,39]: β-lactams [(amoxicillin/clavulanic acid; AMC, 20/10 µg), aminopenicillins (amoxicillin; A, 30 µg), especially for Staphylococcus aureus strains, markers for methicillin resistance (oxacillin, flucloxacillin)]; glycopeptide (vancomycin; VA, 30 µg); second-generation cephalosporins (cefuroxime; CFX, 30 μg); third-generation cephalosporins (cefotaxime; CFT 30 μg); clindamycin (CLI, 2 μg); aminoglycosides (gentamicin; GEN, 10 μg); macrolides (erythromycin; ERY, 15 μg); tetracycline (TER, 30 μg); fluoroquinolones (ciprofloxacin; CIP, 5 μg); carbapenems (imipenem; IMI, 10 μg); and nitroimidazole (metronidazole; MET, 5 µg). 

Furthermore, utilizing the reference broth microdilution method, we determined the minimum inhibitory concentration (MIC) values of the aforementioned antimicrobial agents against the tested bacterial strains, adhering to the guidelines of EUCAST and CLSI for each plant extract type and reference antimicrobials [39,40,41,42,43].

#### 2.3.2. First Assay for Evaluation of the Antibacterial Activity—Diffusion in Agar Test

We initiated the assessment of the antibacterial efficacy of the studied extracts using disc diffusion in agar assays [40]. Overnight cultures of each bacterial strain were prepared and plated onto agar petri dishes, with specific media selected based on the growth requirements of each strain. Dried Mueller–Hinton agar and Brain Heart Infusion agar (Merck KGaA, Darmstadt, Germany) were employed for the *Staphylococcus* and *Streptococcus* strains, respectively, while Brucella Agar supplemented with 5% sheep blood, hemin, and menadione was utilized for obligate anaerobic isolates.

Sterile filter paper discs (6 mm in diameter, Whatman No. 1) saturated with herb extracts at concentrations ranging from 10% to 100% (*v*/*v*) were carefully placed on the surface of each plate using sterile forceps. Aqueous extracts were diluted with sterile distilled water, while discs loaded with sterile distilled water served as the negative controls. After allowing the plates to incubate at room temperature for 2 h to facilitate bacterial diffusion into the agar media, they were then placed in a 37 °C incubator overnight for *S. aureus* strains, precisely 36 h for Streptococcus strains, and at least 72 h for obligate anaerobic strains. Anaerobic conditions were meticulously maintained throughout the incubation period for obligate anaerobic strains using anaerobic rectangular jars equipped with Anaerocult A gas packs (Merck KGaA, Darmstadt, Germany). At the end of the designated incubation period, the zone of inhibition, commonly referred to as the “halo”, was meticulously assessed, with each experiment meticulously conducted in triplicate.

#### 2.3.3. Second Assay for Evaluation of the Antibacterial Activity—Determination of Minimum Inhibitory Concentration and Minimum Bactericidal Concentration—Microdilution Broth Test

To determine the minimum inhibitory concentration (MIC) using the microdilution broth method in 96-well microplates [1,44,45], plant extracts were initially diluted to 400 mg/mL crude extraction in a 5% aqueous solution of dimethyl sulfoxide (DMSO), except for aqueous extracts, which were diluted in ultrapure water. Serial dilutions were then prepared using ultrapure water at concentrations ranging from 200 mg/mL to 0.0975 mg/mL. The bacterial inoculum was standardized to McFarland scale standard 1, and aerobic or facultative anaerobic strains were inoculated in double-strength Mueller–Hinton broth (MHB), while anaerobic bacteria were cultured in sBHI broth supplemented with 5 μg/mL hemin and 1 μg/mL menadione (sBHI; Oxoid, Hampshire, UK). The incubation conditions followed those detailed in the preceding sections. To validate the procedure, control wells containing only the liquid medium, the medium with inoculum, or chlorhexidine (CHX) 0.2% were included. Following incubation, the liquid media in each well were stained with an aqueous resazurin solution (Sigma-Aldrich, St. Louis, MO, USA) at a concentration of 0.02%. The 96-well microplates were then re-incubated for an additional two hours. The MIC was determined as the lowest concentration where the resazurin staining remained blue, indicating inhibition. A color change to pink–purple or pink indicated resazurin reduction and bacterial growth. All experiments were conducted in triplicate. To ascertain the minimum bactericidal concentration (MBC), a 20 μL aliquot from wells that exhibited no growth or from the well corresponding to the MIC reading was transferred onto plates containing Mueller–Hinton Agar or, for anaerobic bacterial strains, 5% defibrinated sheep blood Brucella agar (Merck KGaA, Darmstadt, Germany) enriched with 5 μg/mL hemin, 1 μg/mL menadione, and 2 g/L yeast. The plates were then incubated at 37 °C for 24 h, 36 h, and 37 h for *S. aureus* strains, facultative anaerobic Streptococcus strains, and obligate anaerobic isolates, respectively. The colony growth on the plates was confirmed at the end of this period. MBC was defined as the lowest extract concentration resulting in complete bacterial elimination.

### 2.4. In Vitro Evaluation of Antibacterial Efficacies—Anti-Biofilm Properties of the Studied Herbal Extractions

To assess the biofilm-forming ability of the bacterial isolates [46,47,48], we utilized a semi-quantitative approach employing collagen type I-coated 96-well flat-bottom microplates (Thermo Scientific™ Nunc™, Waltham, MA, USA). Bacterial cultures were prepared in specific broths tailored to their growth requirements: Trypticase Soy Broth supplemented with 1% glucose (TSBG, Merck KGaA, Darmstadt, Germany) for aerobes or facultative anaerobes, and Tryptic Soy Broth supplemented with yeast extract, L-cysteine hydrochloride, hemin, and menadione for obligate anaerobic isolates. Each well of the microplate received 100 μL of bacterial suspension (adjusted to a turbidity of 10^8^ cfu/mL) and was incubated either aerobically at 37 °C for 24–36 h or anaerobically for a minimum of 48 h. Following incubation, the wells were rinsed twice with phosphate-buffered saline (PBS, pH 7.4) and fixed with absolute methanol. The fixed bacterial cells were then stained with 0.1% crystal violet dye for 30 min, washed to remove excess dye, and solubilized with 33% glacial acetic acid. Absorbance at 595 nm was measured to quantify the attached cells, with the negative control wells containing only TSB. Each experiment was conducted in triplicate, and the average absorbance value was compared with the cut-off value (ODc), set as three standard deviations above the mean OD of the negative control.

Based on these findings, the isolates were categorized as non-biofilm producers (OD ≤ ODc), weak biofilm producers (ODc < OD ≤ 2 × ODc), moderate biofilm producers (2 × ODc < OD ≤ 4 × ODc), or strong biofilm producers (4 × ODc < OD).

To assess the anti-biofilm properties of each studied herbal extract, we followed the procedure outlined just above, with the exception that the bacterial suspension in each well was co-cultured with sub-MIC concentrations of the plant extracts as treatment. Wells without any plant extract served as the control samples. The anti-biofilm activity as a percentage (%) of inhibition was calculated using Equation (1) [49] as follows:

Percentage (%) inhibition = [(OD Negative control − OD Sample)/OD Negative control] × 100 (1)

Based on the results, the tested herbal extracts were classified as: excellent (++++) AB activity (>95% inhibition); very good (+++) AB activity (>95–80% inhibition); good (++) AB activity (>80–50% inhibition); poor (+) AB activity (more than 0–50% inhibition); no (−) AB activity (0% or less). *AB meaning: Anti-biofilm.*

### 2.5. Hemolytic Potential of Tested Plant Extracts in Human Erythrocytes

Blood samples (AB type) were obtained from the local hospital, sourced from blood bags with EDTA, slated for disposal due to incomplete blood collection. The blood was centrifuged at 1500 rpm (15 min at 25 °C) to separate the erythrocytes from the plasma and then washed thrice with 10 mL of PBS (pH of 7.4). The erythrocytes were suspended in PBS, and subsequent steps involved multiple washes with 0.2 M PBS (pH of 7.4) and centrifugation at 300 rpm for 10 min, followed by resuspension in saline solution (0.9% NaCl). After thorough washing, the erythrocytes were diluted 1:100 in PBS of a pH of 7 to yield a 1% erythrocyte suspension.

To assess the hemolytic activity of the extracts, various concentrations ranging from 12.50 to 1000 μg/mL were mixed with NaCl solution (0.85%), and a 2% suspension of human erythrocytes was added. The mixture was then incubated at 37 °C for 60 min. Absorbance was measured over time, and hemolysis rates were calculated as a percentage of total hemolysis after one hour.

A negative control comprising only erythrocytes (500 mL of erythrocyte suspension and 1500 mL of PBS buffer, without extract) represented 0% hemolysis, while a positive control included 1% Triton X-100 (hemolyzing agent), indicating 100% hemolysis [50]. The absorbance of each tube was measured at 540 nm (the absorbance of hemoglobin) using a UV–visible spectrophotometer against a blank containing PBS. The hemolysis percentage was calculated using Equation (2) [51] as follows:

Hemolysis activity of plant extract (%) = (ODtest − ODnegative/ODpositive − ODnegative) × 100(2)

### 2.6. Statistical Analysis

The results are presented as the average of three replications plus–minus the corresponding standard deviation. The various observations between groups (phenolic and flavonoid content of plant extracts, DPPH and FRAP assays in plant extracts, disk diffusion, MIC, MBC, and hemolysis (%) between the various pathogens) were compared using ANOVA followed by Tukey’s HSD post hoc comparison at an alpha of 0.05. The Shapiro–Wilk test was applied to ensure the normal distribution of the data prior to ANOVA. All statistical evaluations were performed with SPSS v28 (IBM Corp. Armonk, NY, USA).

## 3. Results

As shown in Table 2, the most abundant phytochemical compounds were found in the aqueous extracts from both plant species and in enzymatic extract from *O. vulgare*.

**Figure 1:**
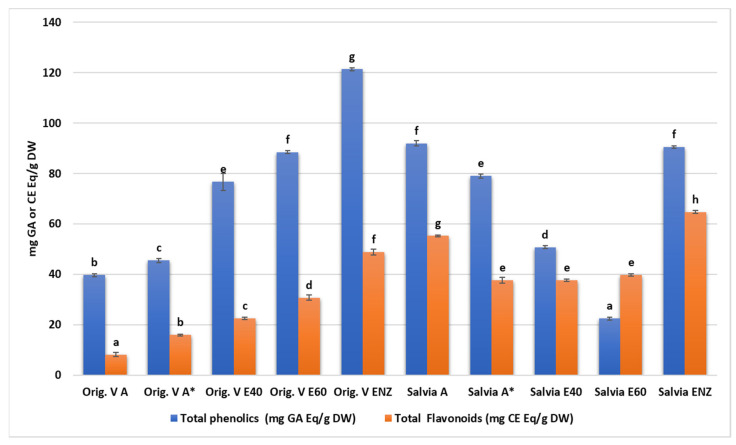
Total phenolic and flavonoid content of *O. vulgare* and *S. triloba* extracts. A*: A solution of an aqueous extract dissolved in a solvent consisting of equal parts (1:1 ratio) boiled water and 95% methanol. Letters above bars indicate similarities or statistically significant differences within total phenolics or flavonoid results (Kruskal–Wallis, *p* < 0.05 with Tukey’s HSD).

All plant extracts were rich in total phenolics with statistically significant differences among their concentration either in each one of the species or between them (Figure 1 and Supplementary Materials Table S1). Among them, higher values were detected in the oregano enzymatic extract (122.4 ± 0.6 mg GAE/g), the salvia aqueous extract (91.99 ± 1.08 mg GAE/g), the salvia enzymatic extract (90.42 ± 0.5 mg GAE/g), and the oregano 60% ethanolic–aqueous extract (88.44 ± 0.6 mg GAE/g). Similarly, the total flavonoid content was increased in salvia and oregano enzymatic extracts (64.75 ± 0.65 mg CE/g and 48.83 ± 1.17 mg CE/g, respectively).

In concentrations above 100 μg/mL, increased scavenging activity was observed in all extracts from both plant species (Table 3). However, the highest values were recorded again in enzymatic fractions with 86.43% and 87.14% for *Salvia* and *Oregano*, respectively.

Similarly, increased plant extracts produced increased reducing power values in FRAP assay (Table 4) with plant extracts being comparable but now statistically significant with the values produced by the two reference compounds. However, in the 25 μg/mL concentration, the absorbance of the oregano enzymatic extract reached 1.93 which was higher than gallic and ascorbic acid.

**Figure 2:**
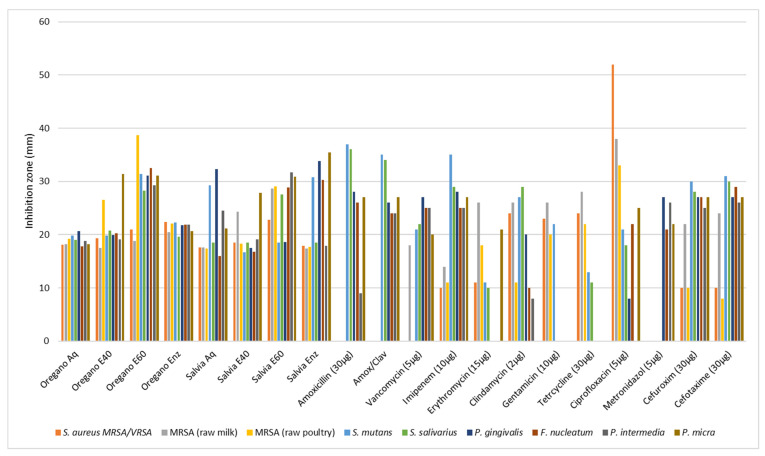
Antimicrobial activity (agar diffusion in mm) of *O. vulgare* and *S. triloba* extracts (Aq, E40, E60, Enz) compared with common antibiotics. Values are means from the best responds (100% disk content) in each experiment.

The report on the antibiotic susceptibility patterns of the studied isolates depicts a varied landscape in terms of the recorded zone of inhibition. Amoxicillin (30 μg) produced zones of inhibition from 9 mm (*P. intermedia*) to 36 or 37 mm (*S. salivarius, S. mutants*). Amoxicillin with clavulanic acid from 24 to 35 mm, vancomycin (30 μg) from 18 to 27 mm, imipenem (10 μg) from 10 to 35 mm, erythromycin (15 μg) from 0 to 26 mm, clindamycin (2 μg) from 0 to 29 mm, gentamicin (10 μg) from 0 to 266 mm, tetracycline (30 μg) from 0 to 28 mm, ciprofloxacin (5 μg) from 0 to 52 mm, metronidazole (5 μg) from 21 to 27 mm, cefuroxime (30 μg) from 10 to 30 mm, and cefotaxime (30 μg) from 8 to 31 mm. Although most of the strains were susceptible or presented a medium susceptibility to common antibiotics, some pathogens (*F. nucleatum*, *P. gingivalis, P. intermedia,* and *P. micra*) were proven multi-resistant in amoxicillin, amoxicillin and clavulanic, vancomycin, imipenem, erythromycin, tetracycline, and ciprofloxacin with MIC values ranging from 0.00025 to 0.128 mg/mL and only *P. micra* against gentamicin reached 0.256 mg/mL (Table 5). The two out the three *S. aureus* isolates were resistant in seven out of ten antibiotics and only the strain isolated from raw milk proved resistant in just two (amoxicillin and amoxicillin/clavulanic acid). In disk diffusion experiments against the oral pathogens, all plant extracts exhibited a variable antibacterial activity with zones of inhibition from 6 to 38 mm (Table 6 and Table 7). In general, the aqueous and ethanolic (E20) *O. vulgare* extracts were comparable with those of *S. triloba* while oregano 60% ethanolic–aqueous and *S. triloba* enzymatic extracts produced wider zones indicating their effectiveness. Most effective was the 60% ethanolic–aqueous extract from oregano in 100% disk content and similarly the 60% ethanolic–aqueous and the enzymatic extracts from *S. triloba*. As shown in Figure 2, concentrated plant extracts produced wider zones of inhibition from most of the antibiotics.

The antibacterial activity of the various plant extracts against oral pathogens as indicated by the minimum inhibitory concentration and minimum bactericidal concentration is presented in Table 8 for *O. vulgare* and in Table 9 for *S. triloba*. All extracts were effective against pathogens since MIC values ranged from 0.4 to 12.5 mg/mL and MBC values from 3.1 to 50 mg/mL. However, the oregano enzymatic and 60% ethanolic–aqueous extracts exhibited the lowest mean MIC values (0.39 ± 0 to 3.12 ± 0 mg/mL) against pathogens while the most effective were also with salvia extracts with mean values from 0.39 ± 0 to 1.56 ± 0 mg/mL. Most sensitive strains in oregano extracts were the MRSA isolated from poultry, *P. gingivalis*, *F. nucleatum*, *P. intermedia* and *P. micra*; meanwhile, in the salvia extracts, the most sensitive strains were MRSA (from raw poultry), *S. mutans*, *S. salivarius*, *P. gingivalis*, *P. intermedia*, *P. micra,* and the reference strain *S. aureus* ATCC 12600 (Ref). Most extracts demonstrated a general antimicrobial effect when compared to the effectiveness of common antibiotics. On comparing the results of Table 5, Table 6, Table 7, Table 8 and Table 9, the higher-in-general-potency of the antibiotics based on the required quantity of substance, with respect to the phytochemical extracts, is obvious. Yet, the term “in general” does not cover all the cases. In many of them, particularly where the conventional antibiotics confront resistance, some phytochemicals show a more effective antibacterial performance. Mult-resistance *S. aureus* from poultry meat shows resistance to imipenem (11 mm inhibition zone) but at the same time is more sensitive to the aquatic and the enzymatic *S. triloba* extracts (for 10%-disc content, 6.4 mm, and 7.8 mm inhibition zones, respectively). *S. mutans* shows resistance to gentamycin (22 mm inhibition zone) but is much more sensitive to the ethanolic 40% *O. vulgare* extract (8.1 mm inhibition zone). Similarly, *P. intermedia* was found resistant to amoxicillin (9 mm inhibition zone) but sensitive to the aquatic extract of *O. vulgare* (6.1 mm inhibition zone). These examples show that phytochemical extracts could potentially be useful in cases of resistance to conventional antibiotics. Another reason that the phytochemical extracts show increased antibacterial potency (mainly regarding MIC values) with respect to the conventional antibiotics is that they are chemically pure substances. In the phytochemical extracts, there are always several different compounds (many of them unknown), in lower concentrations and with unknown synergistic or antagonistic effects, among them. Possibly some of these substances in a chemically pure form may show impressive antibacterial results.

**Figure 3:**
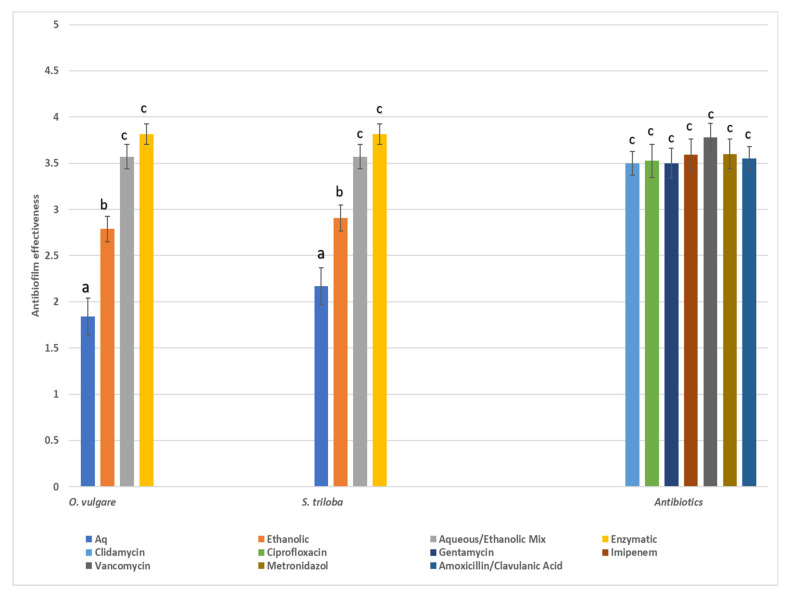
Total median values (score 1: poor, 2: good, 3: very good, 4: excellent) of antibiofilm effectiveness of various types of extracts from the two herbs. Values close to 1 indicate poor biofilm inhibition (0–50%) while values close to 4 indicate excellent biofilm inhibition (>95%), Similar letters above bars indicate no significant difference in antibiofilm effectiveness between similar types of extracts among the various herbal species and antibiotics (Kruskal–Wallis, *p* < 0.05).

The antibiofilm activity was assessed on a four-score scale, categorizing inhibition of biofilm formation as poor (0–50% inhibition), good (50–80% inhibition), very good (80–95% inhibition), and excellent (>95% inhibition). Statistical analysis utilized medians or grouped medians instead of means. In Figure 3, the median scores of various herbal extracts and the scores of the seven antibiotics are presented. Overall, antibiotics demonstrated broad and potent antibiofilm effectiveness against all pathogens tested, with vancomycin showing the highest efficacy. However, herbal extracts from both species, especially the enzymatic and 60% ethanol–aqueous extracts, exhibited significant antibiofilm properties comparable to those of conventional chemical drugs.

In general, low hemolytic activities (0.1% to 9.04%) were observed with plant extract concentrations up to 100 μg/mL for O blood type erythrocytes (Table 10 and Supplementary Materials Figures S1 and S2). However, the most significant activities were observed with *Oregano* E40 and E60 extracts as the concentration further increased. When these two particular extracts were tested at their highest concentration of 1000 μg/mL, hemoglobin release reached considerable percentages of appx. 55% and 83%, respectively, compared to Triton X-100 positive control. No similar activity was detected for the *S. triloba* extracts.

## 4. Discussion

In our study, qualitative phytochemical assays revealed rich profiles in flavonoid, tannins, glycosides, and terpenoids in *O. vulgare* samples, present across all examined extracts. Alkaloids and steroids were exclusively detected in aqueous and enzymatic extracts, respectively [52]. Conversely, *S. triloba* exhibited similar findings, except for the absence of glycosides in enzymatic extracts and no steroids were isolated from any extract. Alkaloids and anthracocyanins were exclusively isolated from the aqueous extract in *S. triloba*, while anthraquinones were only detected in *O. vulgare’s* enzymatic extract. Aqueous extraction effectively retrieved most phytochemicals in both plants, with enzymatic extraction notably enhancing the appearance of certain compounds, particularly in oregano [53]. These results reveal the multifaceted influence of extraction methods, analytical techniques, and plant characteristics on phytochemical profiles [54,55,56,57,58,59]. Additionally, *O. vulgare* essential oil contains a diverse array of terpenes, while *S. triloba oil* is rich in bicyclic oxygenated monoterpenes. Both plants’ infusions and alcoholic extracts exhibit significant polyphenol and flavonoid content, along with di- and triterpenoids [60,61,62].

### 4.1. Pattern of Total Phenolics and Flavonoids Content

In *O. vulgare*, significant variability in total polyphenol content (TPC) was observed among different extracts, with the enzymatic extract showing the highest TPC followed by various ethanol extracts and finally, the aqueous extract, which had the lowest concentration. Similar trends were noted for flavonoid values. These TPC values are consistent with findings from other researchers. For instance, Jafari Khorsand et al. [63] reported TPC ranging from 20.3 to 35.5 mg GAEs g^−1^ DW, while Yan et al. [64], noted values of 79–147 mg GAEs g^−1^ DW in the hydro-methanolic extract of *O. vulgare*. Finally, Bower et al. reported a notable total phenolic content of 430 µg of GAEs mg^−1^ DW for the methanolic extract of *O. vulgare* leaves [62]. Concerning *S. triloba*, higher TPC values were observed in the aqueous and enzymatic extracts compared to ethanol extracts. Researchers calculated TPC and total flavonoid content (TFC) in *S. officinalis* and *S. triloba* extracts, with values ranging from 193.50 ± 8.22 to 203.01 ± 7.85 GAEs mg/g extract for TPC and 71.51 ± 1.88 to 78.84 ± 8.76 QE mg/g extract for TFC [65]. Furthermore, ultrasound-assisted extraction (UAE) extracts exhibited significantly higher values compared to pressurized liquid extraction (PLE) extracts for all photometric determinations. Aqueous methanol mixtures were found to be more effective for UAE extraction of phenolics and flavonoids compared to pure water or methanol [66].

### 4.2. Evaluation of Antioxidant Capacity: DPPH Assay and Ferric-Reducing Assay Power (FRAP)

The DPPH assay is commonly used to evaluate the antioxidant properties of plant extracts, providing insights into their phenolic and flavonoid content [67]. Our study confirmed a dose-dependent scavenging activity, with both *Salvia* and *Oregano* showing enzymatic fractions exhibiting the highest activity. A cut-off concentration limit of 50 μg/mL marks the onset of scavenging activity toward DPPH, with percentages reaching 86.43% and 87.14% for *Salvia* and *Oregano*, respectively, at concentrations of 500 μg/mL (Table 3). This trend persisted across ethanolic and aqueous extracts, with the lowest activity observed in the latter. This indicates that variations in the free radical scavenging activity of extracts are strongly linked to both their chemical composition and the levels of total phenols and flavonoids, which are also concentration-dependent [68,69]. High levels of total phenolic content (TPC) and total flavonoid content (TFC) were observed in *Oregano* extracts, supporting previous research suggesting a correlation between antioxidant activity and phenolic/flavonoid levels [70,71]. In the case of *S. triloba*, high TPC and TFC levels were detected in both aqueous and enzymatic fractions, although aqueous fractions displayed lower DPPH radical neutralization. These variations may be attributed to the unique antioxidant composition within the plant [59]. Consistent with previous studies, *Salvia* extracts exhibited dose-dependent antioxidant activity in the DPPH test [59,65]. The research of Jan et al. [64] also demonstrated dose-dependent antioxidant activity in *O. vulgare* extracts, with higher IC50 values observed in wild accessions compared to cultivated ones. FRAP assay results mirrored these findings, with increased extract concentrations correlating with a higher reducing power (Table 5). Notably, in our study, *Oregano*’s enzymatic extract exhibited superior antioxidant activity compared to the reference compounds. Overall, our findings emphasize the significant antioxidant potential of the extracts tested, irrespective of extraction methods. The capacity of constituents to reduce is a key indicator of their potential antioxidant activity. The ferric-reducing antioxidant power (FRAP) assay relies on the transition from yellow to green, which reflects the sample’s ability to reduce a Fe^3+^/ferricyanide complex to Fe^2+^. Subsequently, the percentage of Fe^2+^ reduced can be quantified by measuring the absorbance at 700 nm [72]. In conclusion, our research findings indicate that regardless of the differences in extraction methods, all types of extracts demonstrated significant antioxidant capacity.

### 4.3. Antimicrobial and Anti-Biofilm Activity of the Studied Extracts

#### 4.3.1. *O. vulgare* Performance

While no distinct antibacterial pattern was discerned, all plant extracts exhibited variable antibacterial activity, with zones of inhibition ranging from 6 to 38.7 mm. The most effective extract was the 60% ethanolic–aqueous extract from oregano at 100% disk content. Compared to synthetic antibiotics, the plant extracts generally produced wider zones of inhibition, particularly when compared to erythromycin. The analyzed extracts exhibited zones of inhibition against the pathogen *S. aureus*, methicillin, and vancomycin, with resistance ranging from approximately 15 to 20 mm. Notably, the ethanol 60% extract displayed the strongest inhibitory properties, followed by the enzymatic extract, the ethanol 40% extract, and the aqueous extract. Analyzing the inhibition zones for all tested microorganisms revealed that extracts with concentrations exceeding 50% exhibited satisfactory inhibition results, spanning from approximately 15.2 mm to 38.7 mm. Given the observed variation, it could be argued that the extracts possess an antibiotic activity akin to pharmaceutical substances. However, since the extracts contain unidentified substances in unspecified concentrations, their effect might be a synergistic or even an antagonistic effect. In the latter scenario, there is a possibility of a potent substance being inhibited by another. This presents a significant limitation of this study. All extracts in our study showed effectiveness against the pathogens, with MIC values ranging from 0.4 to 12.5 mg/mL and MBC values from 3.1 to 50 mg/mL. In particular, the enzymatic and 60% ethanolic–aqueous extracts of oregano displayed the lowest mean MIC values (ranging from 0.39 ± 0 to 3.12 ± 0 mg/mL) against the tested pathogens. Among the strains, MRSA isolated from poultry, *P. gingivalis*, *F. nucleatum*, *P. intermedia*, and *P. micra* were the most sensitive to oregano extracts. The MIC results complement the earlier findings on the minimum zone of inhibition, providing a more detailed assessment of the inhibitory potential of the extracts under study. Once again, the 60% ethanol extract stood out as a potent inhibitor, displaying strong inhibitory capacity even at minimal concentrations. Following closely was the enzymatic extract, while the aqueous extract ranked lowest among the four, although it exhibited a remarkably low concentration requirement for disruption. Ultimately, it becomes apparent that most extracts exhibited comparable or even stronger antimicrobial efficacy when contrasted with the effectiveness of conventional antibiotics. Idir et al. (2022) investigated different extracts (ethanolic 95% and aqueous) of *O. vulgare* against a wide range of oral cavity pathogens, including several *Streptococcus* species. They reported inhibition zones ranging from 8 to 15 mm and MIC values between 1.563 and 12.5 mg/mL [21]. The same researchers noted that, in addition to their antimicrobial effectiveness, *O. vulgare* extracts possess other beneficial properties, such as inhibiting glucosyltransferase activity, reducing acidogenesis, and downregulating the expression of multiple virulence-associated genes in treated samples, as reported in previous investigations [21].

The antibiofilm activity was assessed using a four-point scoring system based on the inhibition of biofilm formation: poor (0–50% inhibition), good (50–80% inhibition), very good (80–95% inhibition), and excellent (>95% inhibition), and the results, along with the scores of the seven antibiotics, are depicted in Figure 3. In general, antibiotic substances displayed broad and potent antibiofilm efficacy against all pathogens, with vancomycin demonstrating the highest effectiveness. Nevertheless, the extracts derived from *O. vulgare*, especially the enzymatic and 60% ethanol–aqueous extracts, showcased significant antibiofilm properties comparable to those of conventional pharmaceuticals. The aforementioned findings corroborate the previous literature suggesting that ethanolic extracts demonstrate superior antimicrobial activity compared to aqueous extracts against a diverse array of oral pathogens, including *Streptococcus*, *Enterococcus*, and *Lacticasei bacillus* [21]. Moreover, researchers have documented that the methanol extract of *O. vulgare* effectively eradicated tested oral bacteria, exhibiting minimum bactericidal concentration (MBC) values ranging from 0.30 mg/mL for *P. micra* to 5.00 mg/mL for *E. faecalis*, *S. mutans*, and *S. sobrinus*. The most pronounced inhibitory effects were observed at an *O. vulgare* concentration of 0.30 mg/mL for *P. gingivalis* and *P. micra*, and 0.60 mg/mL for *S. oralis*, respectively [73]. Considering the commendable activity against biofilm production exhibited by both enzymatic and ethanol extracts, it is evident that these extracts hold promise and warrant further investigation.

#### 4.3.2. *S. tribola* Performance

This study investigated the antimicrobial efficacy of ethanolic (40% and 60%), aqueous, and enzymatic extracts of *S. triloba* against both oral and non-oral pathogens. Our results highlight the significant zones of inhibition, particularly with the ethanolic 60% extract, showing values such as 29.9 mm against MRSA, 30.9 ± 0.3 mm against *P. micra*, and 28.9 mm against *F. nucleatum*. The ethanolic extract exhibited robust antibacterial activity, closely followed by the enzymatic extract. Regarding MIC values, the 60% ethanolic extract displayed exceptionally low values, with the 40% ethanolic extract in proximity. Notably, the aqueous extract showed significant variation among different microorganisms, with a MIC value as low as 0.8 for *P. gingivalis*. The findings regarding biofilm inhibition closely resemble those seen with the oregano plant, with both the enzymatic and 60% ethanolic extracts showing impressive outcomes, akin to antibiotics. Overall, all extracts demonstrate excellent antibiofilm activity against the tested isolates. Previous studies have primarily focused on essential oils or highly concentrated ethanol extracts, whereas our investigation adopts a broader approach by encompassing various types of extracts and a range of pathogens from both oral and food sources. Although existing studies mainly examined *Salvia officinalis*, we aimed to align our experimental protocol as closely as possible. Popa et al. [74] for example, investigated the antimicrobial effects of volatile oils from *S. officinalis* against bacterial strains collected from individuals with oral diseases, with MIC values ranging between 0.00 and 45.9 mg/mL. Other studies reported MIC values for essential oils from *S. officinalis* and *S. triloba* against foodborne bacteria, ranging from 0.3 to 10.0 mg/mL [75]. Additionally, inhibition zones against *S. aureus* were reported for various extracts from *S. fruticosa,* with MIC values ranging from 50 to 15 mg/mL [76]. Evaluation of the glycolic extract of *S. officinalis* against *S. mutans* and other bacterial species revealed effective elimination at a concentration of 50.0 mg/mL [77].

### 4.4. Hemolytic Activity

The in vitro hemolytic test was commonly used in the pharmaceutical industry to screen therapies throughout the early stages of clinical development [66,78]. The occurrence of erythrocyte hemolysis could be an indicator of cell oxidative damage that leads to cell death. Additionally, the lysis of erythrocyte membranes is a common side effect of chemotherapeutic drugs. The co-administration of plant-derived agents is among the potential strategies to mitigate chemotherapeutic-induced hemolysis [79]. A study conducted in vitro cytotoxicity testing based on the hemolysis of type “AB” human blood cells exposed to various concentrations of plant extracts from *O. vulgare* and *S. triloba*. Results were compared to negative and total hemolysis controls. Low hemolytic activities (0.1% to 9.04%) were observed with plant extract concentrations up to 100 μg/mL, but higher activities were noted with *Oregano* E40 and E60 extracts as concentrations increased, reaching approximately 55% and 83%, respectively, at 1000 μg/mL. Clear distinctions were observed in the hemolytic activity of *O. vulgare* extracts, with ethanolic and enzymatic extracts exhibiting higher activity compared to the aqueous extract. On one end of the spectrum are the ethanolic and enzymatic extracts, while on the other end lies the aqueous extract, which demonstrates minimal hemolytic activity even at a concentration of 1000 µg/mL, showing only 10% activity. Across all examined extracts, hemolytic activity remains below 10% up to a concentration of 100 µg/mL, with specific values noted as 5.4%, 6.04%, 7.03%, and 9.04% for the aqueous, E40, E60, and ENZ extracts, respectively. The researchers demonstrated minimal hemolytic activity, remaining below 30% at a concentration of 100 mg/mL, suggesting potential safety when testing the water–ethanol solvent mixture extract of *Origanum grosii* (*O. grosii*).

In our current investigation, *S. triloba* extracts displayed minimal hemolytic activity across all tested concentrations. These findings indicate that S. triloba extracts do not induce hemolysis of red blood cells, suggesting their non-toxic properties. Consistent with our results, Saleh et al. reported that the acetone extract of *S. triloba* exhibited minimal hemolytic effects, with values of 1.282% and 3.157% observed for concentrations of 54.51 µg/mL and 190 µg/mL, respectively, which closely aligns with our findings [80]. However, caution should be exercised when consuming potentially toxic substances such as plants to safeguard health. Additionally, based on the blood group system, it can be indirectly hypothesized that observed hemolysis in blood type “AB” may be due to the presence of antigen A or B on the cell membrane [50,81]. 

### 4.5. General Aspects

The oral cavity should be approached as a highly intricate multi-ecosystem hosting diverse microbial communities. Its distinctive anatomical features and connection to the external environment, primarily through dietary intake, render it a specialized ecological niche for the “oral cavity-dependent” microbiome [82]. Both internal and external factors influence the oral microbiota, maintaining a dynamic equilibrium. A disruption in this balance can alter the microbial composition, leading to oral and potentially systemic diseases. Amidst the current antimicrobial resistance crisis, there is a resurgence in interest in natural plant-derived products, which serve as abundant sources of bioactive substances primarily with antimicrobial properties. However, when these compounds also demonstrate additional biological actions such as antioxidant and anti-inflammatory effects, they epitomize a holistic and comprehensive approach to addressing various diseases. The current study attempted to investigate different types of extracts of two plants (wild ecotypes), *O. vulgare* and *S. triloba*, against a diverse range of isolates found in the oral cavity. These isolates include *F. nucleatum*, *S. salivarius*, *S. mutans*, *P. gingivalis, P. micra*, and *P. intermedia*. Additionally, this study examined isolates, such as multi-antibiotic-resistant *S. aureus*, which may enter the oral cavity through food sources. The significance of the mentioned microorganisms in both oral and systemic health cannot be overstated due to their distinct characteristics. *F. nucleatum*, regarded as a pivotal “bridge” organism, possesses the ability to adhere to various cell types, acting as a conduit for early colonizers such as Streptococcus [83,84]. While *S. mutans* does not act in isolation, it plays a primary role in dental caries development by forming biofilm-based dental plaque on tooth surfaces. This bacterium also produces multiple adhesive proteins that promote plaque formation and subsequent caries. Moreover, *S. mutans* collaborates with other acidogenic and aciduric species, thriving in the acidic, EPS-rich environments it helps establish, thereby exacerbating dental decay [85,86]. Furthermore, inflammation’s role in dysbiosis is underscored by periodontitis-associated bacteria like *P. gingivalis*, which can modulate the host’s immune response [87]. Lastly, emerging research indicates an association between *P. micra* [88] and immune responses in colorectal cancer (CRC), while the influence of *F. nucleatum* and *P. gingivalis* in the carcinogenic process is considered undeniable [89,90,91]. Therefore, it is crucial to prioritize understanding and uncovering substances and mechanisms that operate within the triad of antioxidant, antibacterial, and anti-inflammatory actions, rather than solely concentrating on one pathway.

Food pathogens such as *S. aureus*, are an endless threat to public health, causing significant numbers of illness and deaths every year worldwide [91]. Food safety is thus an important issue for the food industry which is constantly seeking methods to secure its products from hazardous microorganisms. Many of the various chemical substances that have been used as food preservatives are considered detrimental to human health and include substances such as sodium nitrite [92]. Consumers are very reluctant to accept synthetic food preservatives while they are much more tolerant of natural substances [93,94]. Phytochemicals could be a viable alternative to this problem [95,96] and could be incorporated in the food matrix, added into the packaging material, or coat the food [97,98]. All historical civilizations have used phytochemicals for medical, cosmetic, and culinary purposes and this ethnopharmacological data has been passed to us as a robust corpus of empirical knowledge to exploit [99]. Of course, phytochemicals should pass the safety tests as must every other compound intended for the food industry.

In the backdrop of the preceding discussion, it is worth highlighting that research has uncovered the abundance of specific active ingredients in Oregano and Salvia plants, attributed to their anti-inflammatory, antioxidant, and antibacterial properties, whether they act individually or synergistically. These include tannins, flavonoids, triterpenes, and phenols such as rosmarinic acid, linalool, thymol, carvacrol, caffeic acid, carnosic acid, carnosol, salvianolic acid B, and scutellarin [49,100]. The analyzed samples revealed abundant bioactive compounds, such as phenolics and flavonoids, contributing to a robust antioxidant effect. Furthermore, they exhibited notable activity against biofilm formation and demonstrated significant antibacterial efficacy, frequently outperforming conventional antibiotics.

While this study provides insights into the phytochemical composition and bioactivities, limitations include its focus on only two plant species, methodological constraints, and a lack of mechanistic insights. Future research should address these limitations through standardized methodologies and in-depth mechanistic studies using in vivo models or clinical trials. Despite its limitations, this study highlights the antioxidant, antibacterial, and anti-biofilm properties of the extracts, suggesting their potential in oral care. However, sustainability concerns regarding habitat destruction and biodiversity loss due to extraction processes warrant further exploration of eco-friendly alternatives, including synthetic or bioengineered compounds [101,102].

## 5. Conclusions

1. This study reports on the multifactorial dependence of phytochemical isolation on factors such as extraction methods, analytical techniques, and plant species’ unique characteristics. Despite variations in extraction methodologies, all types of extracts demonstrated significant antioxidant capacity, highlighting the potential health benefits of these plants.
2. Additionally, the extracts exhibited promising antibacterial activity against a diverse range of oral pathogens, including antibiotic-resistant strains, and showed efficacy in inhibiting oral biofilm formation. While this study has limitations, such as its focus on only two plant species and methodological constraints, it provides a foundation for future research to explore the mechanisms underlying the observed bioactivities and validate the efficacy and safety of plant-derived treatments through in vivo models or clinical trials.
3. The extracts from both plants showed an exceptional antibacterial action against the two *S. aureus* strains which originated from poultry meat and raw milk. This finding is essential for the future development of food preservatives based on these extracts.

Overall, the findings suggest that *O. vulgare* and *S. triloba* extracts contain bioactive compounds with potential therapeutic value, warranting further investigation in the field of natural product pharmacology and therapeutics.

## Figures and Tables

**Table 1:** Strains of pathogenic bacteria that were used in this study.

Isolate	Source	Characterization Based on Gram Staining	Oxygen Requirements for Growth/ Growth Conditions Used in the Present Study
*Streptococcus mutans*	Oral cavity	Gram ^+^	Facultatively anaerobic/Growth under anaerobic conditions
*Streptococcus salivarius*	Oral cavity	Gram ^+^	Facultatively anaerobic/Growth under anaerobic conditions
*Fusobacterium nucleatum*	Oral cavity	Gram ^−^	Obligate anaerobic/ Growth under anaerobic conditions
*Porphyromonas gingivalis*	Oral cavity	Gram ^−^	Obligate anaerobic/ Growth under anaerobic conditions
*Prevotella intermedia*	Oral cavity	Gram ^−^	Obligate anaerobic/ Growth under anaerobic conditions
*Parvimonas micra*	Oral cavity	Gram ^+^	Obligate anaerobic/ Growth under anaerobic conditions
*Staphylococcus aureus, Methicillin,* and *vancomycin-resistant*	Dental caries area	Gram ^+^	Best in the presence of oxygen-rich environment/ Growth under aerobic conditions
*Staphylococcus aureus,* Methicillin-Resistant	Raw milk	Gram ^+^	Best in the presence of oxygen-rich environment/ Growth under aerobic conditions
*Staphylococcus aureus,* Methicillin-Resistant	Poultry meat	Gram ^+^	Best in the presence of oxygen-rich environment/ Growth under aerobic conditions
*Staphylococcus aureus* subsp. *aureus*	Reference strain ATCC 6538		

**Table 2:** Phytochemical constituents present in individual plant extracts.

Phytochemical Compounds	Plant Extracts							
	*Origanum vulgare*				*Salvia triloba*			
	A	Ε40	Ε60	ΕΝΖ	A	Ε40	Ε60	ΕΝΖ
Alkaloids	+	-	-	+	+	-	-	-
Anthraquinones	-	-	-	+	+	-	-	-
Terpenoids (Salkowski’s test)	+	+	+	+	+	+	+	+
Steroids	+	-	-	+	-	-	-	-
Saponins	-	+	+	-	+	+	-	-
Flavonoids (alkaline reagent test)	+	+	+	+	+	+	+	+
Tannins (ferric chloride test)	+	+	+	-	+	+	+	+
Glycosides (Keller–Kiliani test)	+	+	+	+	+	+	+	-

A—aqueous extract; E40—ethanolic extract (40% v/v aqueous ethanol); E60—ethanolic extract (60% *v*/*v* aqueous-ethanol); ENZ—enzymatic extract; +: positive detection, -: not detected.

**Table 3:** Percentage of neutralization of the DPPH radical by plant extracts in the DPPH assay.

Plant Extracts	Concentration (μg/mL)					
	1	10	50	100	300	500
Oregano A	3.42 ± 0.33 ^bc^	10.57 ± 0.13 ^c^	33.34 ± 0.58 ^b^	38.55 ± 0.21 ^b^	52.24 ± 0.22 ^a^	59.32 ± 0.85 ^a^
Oregano A*	5.28 ± 0.1 ^d^	12.18 ± 0.42 ^d^	40.86 ± 0.02 ^e^	45.26 ± 0.53 ^c^	59.52 ± 0.4 ^b^	62.92 ± 0.35 ^b^
Oregano E40	4.12 ± 0.11 ^cf^	12.19 ± 0.23 ^d^	50.46 ± 0.34 ^g^	70.48 ± 0.07 ^g^	74.5 ± 0.39 ^e^	77.33 ± 0.11 ^d^
Oregano E60	12.37 ± 0.28 ^g^	18.06 ± 0.19 ^f^	55.24 ± 0.21 ^h^	76.79 ± 0.22 ^h^	80.7 ± 0.17 ^f^	81.36 ± 0.25 ^e^
Oregano ENZ	11.6 ± 0.45 ^ef^	14.32 ± 0.34 ^e^	57.99 ± 0.22 ^i^	78.77 ± 0.17 ^i^	82.44 ± 0.42 ^g^	86.43 ± 0.42 ^g^
Salvia A	11.5 ± 0.39 ^e^	14.12 ± 0.12 ^e^	39.53 ± 0.45 ^d^	60.53 ± 0.33 ^f^	82.21 ± 0.32 ^g^	84.24 ± 0.29 ^f^
Salvia A*	3.42 ± 0.16 ^bc^	9.36 ± 0.27 ^b^	32.38 ± 0.44 ^b^	48.43 ± 0.34 ^d^	80.13 ± 0.29 ^f^	82.38 ± 0.44 ^e^
Salvia E40	2.63 ± 0.2 ^b^	10.22 ± 0.19 ^c^	35.27 ± 0.22 ^c^	48.36 ± 0.39 ^d^	72.37 ± 0.26 ^d^	74.49 ± 0.42 ^c^
Salvia E60	1.37 ± 0.41 ^a^	6.33 ± 0.25 ^a^	22.38 ± 0.41 ^a^	30.31 ± 0.23 ^a^	54.97 ± 0.44 ^b^	60.53 ± 0.41 ^a^
Salvia ENZ	12.71 ± 0.24 ^g^	17.37 ± 0.42 ^f^	42.73 ± 0.25 ^f^	59.25 ± 0.18 ^e^	85.46 ± 0.21 ^h^	87.14 ± 0.71 ^g^

A*: A solution of an aqueous extract dissolved in a solvent consisting of equal parts (1:1 ratio) boiled water and 95% methanol. Values are the mean of three replicates. Different superscript letters in columns denote statistical differences among the various extracts in each plant (Kruskal–Wallis, *p* < 0.05 with Tukey’s HSD).

**Table 4:** Reducing power of different concentrations of extracts from *O. vulgare* and *S. triloba* as evaluated by the FRAP assay.

Plant Extracts	Concentration (μg/mL)					
	2.5	5	10	15	20	25
Oregano A	0.15 ± 0 ^a^	0.21 ± 0 ^a^	0.31 ± 0 ^a^	0.57 ± 0 ^a^	0.77 ± 0 ^a^	0.85 ± 0 ^a^
Oregano A*	0.21 ± 0 ^c^	0.40 ± 0 ^c^	0.55 ± 0 ^f^	0.81 ± 0 ^c^	1.01 ± 0 ^c^	1.21 ± 0 ^d^
Oregano E40	0.22 ± 0 ^d^	0.49 ± 0 ^g^	0.75 ± 0 ^h^	1.28 ± 0 ^h^	1.44 ± 0 ^g^	1.62 ± 0 ^f^
Oregano E60	0.24 ± 0 ^c^	0.55 ± 0 ^h^	0.83 ± 0 ^i^	1.34 ± 0 ^i^	1.55 ± 0 ^h^	1.77 ± 0 ^g^
Oregano ENZ	0.23 ± 0 ^c^	0.58 ± 0 ^i^	0.99 ± 0 ^j^	1.48 ± 0 ^j^	1.65 ± 0 ^i^	1.93 ± 0 ^i^
Salvia A	0.24 ± 0 ^c^	0.37 ± 0 ^d^	0.44 ± 0 ^bc^	0.78 ± 0 ^d^	0.98 ± 0 ^d^	1.33 ± 0 ^c^
Salvia A*	0.18 ± 0 ^b^	0.32 ± 0 ^c^	0.54 ± 0 ^f^	0.71 ± 0 ^c^	0.86 ± 0 ^bc^	1.11 ± 0 ^c^
Salvia E40	0.19 ± 0 ^b^	0.31 ± 0 ^c^	0.48 ± 0 ^d^	0.68 ± 0 ^b^	0.85 ± 0 ^b^	1.09 ± 0 ^b^
Salvia E60	0.18 ± 0 ^b^	0.28 ± 0 ^b^	0.38 ± 0 ^b^	0.70 ± 0 ^c^	0.85 ± 0 ^b^	1.08 ± 0 ^b^
Salvia ENZ	0.26 ± 0 ^f^	0.38 ± 0 ^d^	0.51 ± 0 ^c^	0.88 ± 0 ^f^	1.31 ± 0 ^f^	1.76 ± 0 ^g^
Reference						
Gallic acid (GA)	0.18 ± 0.0 ^b^	0.43 ± 0 ^f^	1.81 ± 0 ^k^	1.85 ± 0 ^k^	1.88 ± 0 ^j^	1.88 ± 0 ^h^
Ascorbic acid (AA)	0.19 ± 0.0 ^b^	0.42 ± 0 ^f^	0.68 ± 0 ^g^	1.12 ± 0 ^g^	1.65 ± 0 ^i^	1.88 ± 0 ^h^

A*: A solution of an aqueous extract dissolved in a solvent consisting of equal parts (1:1 ratio) boiled water and 95% methanol. Values are the mean of three replicates. Different superscript letters in columns denote statistical differences among the various extracts in each plant (Kruskal–Wallis, *p* < 0.05 with Tukey’s HSD).

**Table 5:** Phenotypically antimicrobial susceptibility of the studied pathogens against common antibiotic agents. For interpretation purposes, values indicating resistance (R) are shown in red, sensitivity (S) in blue, and intermediate (I) in green. Interpretation values according to the guidelines of EUCAST and CLSI [39,40,41,42,43].

Bacterial Strain	Antimicrobial Agents																							
	Amoxicillin		Amoxicillin/ Clavulanic Acid		Vancomycin		Imipenem		Erythromycin		Clindamycin		Gentamicin		Tetracycline		Ciprofloxacin		Metronidazole		Cefuroxime		Cefotaxime	
	mm	MIC mg/mL	mm	MIC mg/mL	mm	MIC mg/mL	mm	MIC mg/mL	mm	MIC mg/mL	mm	MIC mg/mL	mm	MIC mg/mL	mm	MIC mg/mL	mm	MIC mg/mL	mm	MIC mg/mL	mm	MIC mg/mL	mm	MIC mg/mL
*Staphylococcus aureus* subsp. *aureus*, methicillin, and vancomycin-resistant	0	0.128	0	0.0128	0	0.064	10	0.032	11	0.032	24	0.00025	23	0.0005	24	0.001	52	0.00125	- *	-	10	0.064	10	0.064
Methicillin-Resistant *S. aureus*, from raw milk	0	0.064	0	0.064	18	0.002	14	0.004	26	0.0005	26	0.00025	26	0.0005	28	0.0005	38	0.00125	-	-	22	0.002	24	0.006
Methicillin-Resistant *S. aureus*, from raw poultry	0	0.128	0	0.128	0	0.032	11	0.032	18	0.008	11	0.008	20	0.001	22	0.001	33	0.00125	-	-	10	0.032	8	0.032
*Streptococcus mutans*	37	0.00025	35	0.000125	21	0.0005	35	0.0005	11	0.004	27	0.000125	22	0.024	13	0.008	21	0.0005	-	-	30	0.00025	31	0.0005
*Streptococcus salivarius*	36	0.000125	34	0.000125	22	0.0005	29	0.001	10	0.016	29	0.000125	0	0.096	11	0.010	18	0.001	-	-	28	0.0005	30	0.0005
*Fusobacterium nucleatum*	26	0.00025	24	0.004/0.002	25	0.00025	25	0.00025	0	0.064	10	0.016	0	0.128	0	0.032	22	0.001	21	0.001	27	0.004	29	0.004
*Porphyromonas gingivalis*	28	0.00025	26	0.004/0.002	27	0.00025	28	0.00025	0	0.064	20	0.001	0	0.128	0	0.032	8	0.008	27	0.0005	27	0.002	27	0.004
*Prevotella intermedia*	9	0.016	24	0.004/0.002	25	0.00025	25	0.00025	0	0.064	8	0.032	0	0.128	0	0.032	0	0.016	26	0.0005	25	0.002	26	0.002
*Parvimonas micra*	27	0.0005	27	0.002/0.001	20	0.0025	27	0.00015	21	0.004	0	0.032	0	0.256	0	0.032	25	0.0005	22	0.0005	27	0.00025	27	0.0005

*: did not perform.

**Table 6:** Antibacterial activities (disk diffusion) of various concentrations (10, 20, 50, and 100%) of aqueous (A), ethanolic (E40 and E60) and enzymatic (Enz) extracts from *O. vulgare* against oral/food origin pathogens and a reference strain.

Pathogen	Disk Content (%)	A (mm)	E40 (mm)	E60 (mm)	Enz (mm)
*S. aureus* MRSA/VRSA	10	6 ± 0.1 ^a^	7.5 ± 0.5 ^a^	9.7 ± 0.6 ^ab^	11.5 ± 0.4 ^d^
	20	13.8 ± 0.8 ^ef^	10 ± 0.6 ^ab^	14.7 ± 0.5 ^b^	15 ± 0.5 ^e^
	50	15.7 ± 0.5 ^ab^	16.2 ± 0.6 ^b^	18.3 ± 0.7 ^a^	19.1 ± 1.1 ^b^
	100	18.1 ± 0.3 ^ab^	19.3 ± 0.6 ^bc^	21 ± 0.1 ^b^	22.4 ± 0.1 ^b^
MRSA (raw milk)	10	6.5 ± 0.3 ^a^	8.4 ± 0.3 ^a^	10.8 ± 0.6 ^bc^	8.7 ± 0.5 ^ab^
	20	10.8 ± 0.3 ^bc^	10.4 ± 0.3 ^ab^	15.1 ± 0.4 ^b^	15.3 ± 0.9 ^e^
	50	9.7 ± 6.8 ^a^	14.1 ± 0.1 ^a^	17.6 ± 0.5 ^a^	19 ± 0.9 ^b^
	100	18.2 ± 0.4 ^ab^	17.5 ± 0.5 ^a^	18.8 ± 0.6 ^a^	20.4 ± 0.4 ^ab^
MRSA (raw poultry)	10	6.9 ± 0.1 ^a^	9.8 ± 0.6 ^bc^	12.2 ± 0.2 ^d^	8 ± 0.5 ^a^
	20	11.5 ± 0.4 ^de^	13.2 ± 0.6 ^de^	18.5 ± 0.3 ^c^	15.1 ± 0.4 ^e^
	50	22.9 ± 5.2 ^b^	19.5 ± 0.3 ^d^	27.8 ± 0.9 ^d^	18.7 ± 0.5 ^b^
	100	19.2 ± 0.4 ^cd^	26.5 ± 0.6 ^d^	38.7 ± 0.8 ^f^	22.1 ± 0.5 ^b^
*S. mutans*	10	9.6 ± 0.7 ^c^	8.1 ± 0.3 ^a^	12.1 ± 0.8 ^c^	9.5 ± 0.4 ^b^
	20	12.6 ± 0.4 ^de^	11.2 ± 0.6 ^ab^	19.3 ± 0.5 ^de^	12.4 ± 0.2 ^bc^
	50	17.1 ± 0.4 ^ab^	17.3 ± 0.6 ^cd^	28.6 ± 0.9 ^d^	18.8 ± 0.3 ^b^
	100	19.8 ± 0.5 ^cd^	19.8 ± 0.4 ^bc^	31.4 ± 0.6 ^e^	22.3 ± 2.7 ^b^
*S. salivarius*	10	6.2 ± 0.2 ^a^	8.1 ± 0.2 ^a^	10.7 ± 0.5 ^b^	7.7 ± 0.6 ^a^
	20	9.8 ± 0.3 ^ab^	11.8 ± 0.3 ^bcd^	18.5 ± 0.7 ^cd^	11.5 ± 0.5 ^ab^
	50	15.2 ± 0.4 ^ab^	15.9 ± 0.4 ^b^	25 ± 0.4 ^c^	17 ± 0.1 ^b^
	100	19 ± 0.3 ^cd^	20.7 ± 1.3 ^c^	28.2 ± 0.5 ^c^	19.6 ± 0.5 ^ab^
*P. gingivalis*	10	8.6 ± 0.5 ^bc^	10.7 ± 0.5 ^cd^	12.3 ± 0.4 ^d^	9.7 ± 0.5 ^bc^
	20	13.5 ± 0.4 ^ef^	14.8 ± 0.1 ^e^	19.5 ± 0.6 ^de^	14.1 ± 0.7 ^de^
	50	18.2 ± 0.5 ^b^	19.5 ± 0.3 ^d^	29 ± 0.3 ^d^	19 ± 0.3 ^b^
	100	20.6 ± 0.7 ^d^	19.9 ± 0.1 ^bc^	31.1 ± 0.2 ^e^	21.8 ± 0.4 ^b^
*F. nucleatum*	10	6.1 ± 0.2 ^a^	7.9 ± 0.2 ^a^	13.3 ± 0.2 ^d^	10.1 ± 0.2 ^c^
	20	8.9 ± 0.6 ^a^	14.8 ± 0.2 ^e^	19.1 ± 0.2 ^de^	13.8 ± 0.5 ^cd^
	50	15.3 ± 0.5 ^ab^	18.1 ± 0.7 ^cd^	28.2 ± 0.5 ^d^	18.7 ± 0.7 ^b^
	100	17.8 ± 0.5 ^ab^	20.2 ± 0.3 ^c^	32.5 ± 1.5 ^ef^	21.9 ± 0.3 ^b^
*P. intermedia*	10	6.1 ± 0.1 ^a^	8.6 ± 0.4 ^ab^	10.6 ± 0.2 ^b^	8.4 ± 0.7 ^a^
	20	12.5 ± 0.4 ^ef^	12.4 ± 0.5 ^cd^	17.2 ± 0.5 ^c^	11.2 ± 0.8 ^a^
	50	16.8 ± 0.5 ^ab^	17.1 ± 0.3 ^bc^	21.9 ± 1.8 ^b^	18.1 ± 0.2 ^b^
	100	18.8 ± 0.3 ^abc^	19.1 ± 0 ^bc^	29.3 ± 0.6 ^b^	21.9 ± 0.7 ^b^
*P. micra*	10	6.3 ± 0.6 ^a^	11.7 ± 0.5 ^d^	12.6 ± 0.6 ^d^	9.6 ± 0.5 ^b^
	20	14.8 ± 0.4 ^f^	18.2 ± 0.4 ^f^	20 ± 0.2 ^e^	13.3 ± 0.2 ^cd^
	50	16.9 ± 0.4 ^ab^	24.2 ± 1.1 ^e^	27.9 ± 0.4 ^d^	18 ± 0.2 ^b^
	100	18.2 ± 0.2 ^ab^	31.4 ± 0.4 ^e^	31.1 ± 0.1 ^e^	20.6 ± 0.4 ^ab^
*S. aureus* ATCC 12600 (Ref)	10	8.3 ± 0.5 ^b^	8.1 ± 0.6 ^a^	9.1 ± 0.1 ^a^	10.2 ± 0.3 ^c^
	20	12.8 ± 0.5 ^de^	12.5 ± 1.3 ^c^	12.3 ± 0.6 ^a^	13 ± 0.3 ^bc^
	50	16.6 ± 0.4 ^ab^	17.4 ± 0.6 ^bc^	18 ± 0.3 ^a^	17.8 ± 0.4 ^ab^
	100	17.6 ± 0.5 ^a^	19.1 ± 0.2 ^bc^	18.7 ± 0.5 ^a^	19.2 ± 0.4 ^a^

Values are the mean of three replicates. Different superscript letters in a row indicate significant differences among the various extracts for similar disk content (Kruskal–Wallis with Tukey’s HSD, *p* < 0.05).

**Table 7:** Antibacterial activities (disk diffusion) of various concentrations (10, 20, 50, and 100%) of aqueous (A), ethanolic (E40 and E60), and enzymatic (Enz) extracts from *S. trilova* against oral/food origin pathogens and a reference strain.

Pathogen	Disk Content (%)	A (mm)	E40 (mm)	E60 (mm)	Enz (mm)
*S. aureus* MRSA/VRSA	10	6.7 ± 0.6 ^ab^	10 ± 0.7 ^cd^	10.5 ± 0.5 ^abc^	6 ± 0 ^a^
	20	11.3 ± 0.4 ^bc^	12.6 ± 0.2 ^cd^	15 ± 0.4 ^bc^	8.5 ± 0.6 ^a^
	50	13.4 ± 0.4 ^ac^	17.5 ± 0.5 ^cd^	18.9 ± 0.5 ^bc^	12.2 ± 0.3 ^a^
	100	17.6 ± 0.3 ^abc^	18.5 ± 0.3 ^bc^	22.8 ± 2 ^ab^	17.9 ± 0.4 ^a^
MRSA (raw milk)	10	7.6 ± 0.6 ^ab^	12.5 ± 0.4 ^e^	12.6 ± 0.3 ^d^	7.4 ± 0.5 ^b^
	20	11.9 ± 0.7 ^bc^	15 ± 0.2 ^e^	18.6 ± 0.3 ^ef^	12 ± 0.4 ^c^
	50	14.8 ± 0.5 ^bc^	18 ± 0.3 ^d^	23.2 ± 0.6 ^e^	14.6 ± 0.2 ^b^
	100	17.6 ± 0.3 ^abc^	24.3 ± 0.4 ^e^	28.7 ± 0.6 ^c^	17.4 ± 0.4 ^a^
MRSA (raw poultry)	10	6.4 ± 0.4 ^a^	9.1 ± 0.7 ^ab^	11.4 ± 0.7 ^bcd^	7.8 ± 0.5 ^bc^
	20	10.6 ± 0.1 ^bc^	10.6 ± 0.2 ^a^	16.2 ± 0.6 ^cd^	10.7 ± 0.4 ^b^
	50	14.9 ± 0.2 ^bc^	16.2 ± 0.3 ^bc^	20.6 ± 0.5 ^cd^	15.2 ± 0.4 ^bc^
	100	17.4 ± 0.4 ^bc^	18.3 ± 0.6 ^abc^	29.1 ± 0.8 ^c^	17.7 ± 0.3 ^a^
*S. mutans*	10	10.8 ± 0.8 ^de^	8.6 ± 0.3 ^ab^	9.8 ± 0.4 ^a^	12.1 ± 0.5 ^e^
	20	16.4 ± 0.4 ^f^	10.7 ± 0.3 ^ab^	11.9 ± 0.5 ^a^	17.1 ± 0.2 ^cd^
	50	20.7 ± 0.8 ^g^	15.2 ± 0.3 ^ab^	16.7 ± 0.5 ^ab^	21.5 ± 0.7 ^e^
	100	29.3 ± 0.9 ^f^	16.7 ± 0.2 ^a^	18.5 ± 0.5 ^a^	30.8 ± 0.6 ^b^
*S. salivarius*	10	8.4 ± 0.8 ^bc^	10.8 ± 0.6 ^de^	11.7 ± 0.4 ^cd^	9.3 ± 0.3 ^d^
	20	12.9 ± 0.6 ^de^	13.1 ± 0.8 ^d^	17.4 ± 0.5 ^de^	12.8 ± 0.5 ^b^
	50	16.4 ± 0.5 ^de^	16.3 ± 0.5 ^bc^	22.2 ± 0.7 ^de^	17.8 ± 0.3 ^d^
	100	18.5 ± 0.6 ^cd^	18.5 ± 0.5 ^bc^	27.5 ± 5.5 ^bc^	18.5 ± 0.4 ^a^
*P. gingivalis*	10	11.2 ± 0.8 ^e^	7.6 ± 0.5 ^a^	10.2 ± 0.2 ^ab^	12.2 ± 0.6 ^e^
	20	15.2 ± 0.5 ^f^	11.9 ± 0.3 ^c^	14 ± 0.7 ^b^	18.2 ± 0.3 ^de^
	50	20.6 ± 0.5 ^fg^	14.1 ± 0.3 ^a^	17 ± 0.2 ^ab^	28.1 ± 1 ^g^
	100	32.3 ± 1.9 ^g^	17.5 ± 0.3 ^ab^	18.6 ± 0.4 ^a^	33.8 ± 1.2 ^cd^
*F. nucleatum*	10	6.1 ± 0.1 ^a^	9.8 ± 0.4 ^bcd^	11.9 ± 0.6 ^cd^	9.8 ± 0.4 ^d^
	20	9.3 ± 0.6 ^a^	10.4 ± 0.4 ^a^	17.2 ± 0.2 ^de^	16.7 ± 0.4 ^c^
	50	12.4 ± 0.6 ^a^	13.9 ± 0.2 ^a^	24.1 ± 1.4 ^e^	19.6 ± 0.8 ^d^
	100	16 ± 0.2 ^b^	16.8 ± 0.2 ^a^	28.9 ± 0.9 ^c^	30.3 ± 0.8 ^b^
*P. intermedia*	10	9.6 ± 0.2 ^cde^	8.3 ± 0.6 ^ab^	11.7 ± 0.5 ^cd^	9 ± 0.1 ^cd^
	20	13.7 ± 0.2 ^e^	12 ± 0.4 ^c^	19 ± 0.4 ^f^	12.5 ± 0.7 ^b^
	50	19 ± 0.2 ^ef^	17.3 ± 0.4 ^cd^	28.4 ± 0.6 ^f^	16.7 ± 0.4 ^cd^
	100	24.5 ± 0.5 ^e^	19.1 ± 0.8 ^c^	31.7 ± 0.6 ^c^	17.9 ± 0.1 ^a^
*P. micra*	10	9.5 ± 0.6 ^cd^	10.6 ± 0.6 ^cd^	11 ± 0.5 ^abc^	12 ± 0.5 ^e^
	20	12.4 ± 0.4 ^de^	15.3 ± 0.5 ^e^	16.2 ± 0.3 ^cd^	19.3 ± 0.5 ^e^
	50	17.8 ± 0.9 ^de^	21.6 ± 1.2 ^e^	20.8 ± 0.5 ^de^	29.3 ± 0.5 ^f^
	100	21.2 ± 0.9 ^d^	27.8 ± 1.1 ^f^	30.9 ± 0.3 ^c^	35.4 ± 1.1 ^d^
*S. aureus* ATCC 12600 (Ref)	10	9.4 ± 0.5 ^cd^	10.9 ± 1.1 ^de^	10.5 ± 0.6 ^abc^	11.1 ± 0.1 ^e^
	20	12.9 ± 0.5 ^de^	14.9 ± 0.1 ^e^	18.1 ± 0.6 ^ef^	18 ± 0.2 ^de^
	50	17.5 ± 0.6 ^de^	18.3 ± 1 ^d^	26.7 ± 0.7 ^f^	22.2 ± 1 ^e^
	100	19.8 ± 0.4 ^cd^	21 ± 0.4 ^d^	30.7 ± 0.4 ^c^	31.8 ± 2 ^bc^

Values are the mean of three replicates. Different superscript letters in a row indicate significant differences among the various extracts for similar disk content (Kruskal–Wallis with Tukey’s HSD, *p* < 0.05).

**Table 8:** Minimum inhibitory concentrations (MIC) and minimum bactericidal concentrations (MBC) of the various extracts (A-aqueous; E40 and E60—ethanolic 40% and 60%; and Enz—enzymatic) from *O. vulgare*, against pathogens. Mean values (mg/mL) from three repetitions.

	Minimum Inhibitory Concentration (mg/mL)				Minimum Bactericidal Concentration (mg/mL)			
Pathogen	A	E40	E60	Enz	A	E40	E60	Enz
*S. aureus* MRSA/VRSA	6.3 ± 0 ^b^	3.1 ± 0 ^b^	1.6 ± 0 ^b^	1.6 ± 0 ^b^	12.5 ± 0 ^b^	6.3 ± 0 ^c^	3.1 ± 0 ^b^	1.6 ± 0 ^a^
MRSA (raw milk)	6.3 ± 0 ^b^	6.3 ± 0	3.1 ± 0 ^c^	3.1 ± 0 ^c^	12.5 ± 0 ^b^	12.5 ± 0	12.5 ± 0 ^d^	6.3 ± 0 ^c^
MRSA (raw poultry)	3.1 ± 0 ^a^	0.8 ± 0 ^a^	0.4 ± 0 ^a^	0.8 ± 0 ^a^	6.3 ± 0 ^a^	1.6 ± 0 ^a^	1.6 ± 0 ^a^	1.6 ± 0 ^a^
*S. mutans*	3.1 ± 0 ^b^	3.1 ± 0 ^b^	1.6 ± 0 ^b^	3.1 ± 0 ^c^	6.3 ± 0 ^a^	12.5 ± 0 ^d^	3.1 ± 0 ^b^	6.3 ± 0 ^c^
*S. salivarius*	6.3 ± 0 ^b^	3.1 ± 0 ^b^	1.6 ± 0 ^b^	3.1 ± 0 ^c^	12.5 ± 0 ^b^	6.3 ± 0 ^c^	6.3 ± 0 ^c^	6.3 ± 0 ^c^
*P. gingivalis*	6.3 ± 0 ^b^	6.3 ± 0 ^c^	1.6 ± 0 ^b^	0.8 ± 0 ^a^	25 ± 0 ^c^	12.5 ± 0 ^d^	3.1 ± 0 ^b^	6.3 ± 0 ^c^
*F. nucleatum*	6.3 ± 0 ^b^	3.1 ± 0 ^b^	0.8 ± 0 ^a^	3.1 ± 0 ^c^	25 ± 0 ^c^	12.5 ± 0 ^d^	3.1 ± 0 ^b^	3.1 ± 0 ^b^
*P. intermedia*	6.3 ± 0 ^b^	3.1 ± 0 ^b^	0.8 ± 0 ^a^	3.1 ± 0 ^c^	25 ± 0 ^c^	12.5 ± 0 ^d^	6.3 ± 0 ^c^	12.5 ± 0 ^d^
*P. micra*	12.5 ± 0 ^c^	0.8 ± 0 ^a^	0.8 ± 0 ^a^	1.6 ± 0 ^b^	25 ± 0 ^c^	3.1 ± 0 ^b^	6.3 ± 0 ^c^	6.3 ± 0 ^c^
*S. aureus* ATCC 12600 (Ref)	6.3 ± 0 ^b^	3.1 ± 0 ^b^	3.1 ± 0 ^c^	1.6 ± 0 ^b^	12.5 ± 0 ^b^	12.5 ± 0 ^d^	12.5 ± 0 ^d^	6.3 ± 0 ^c^

Values are the mean of three replicates. Different superscript letters in a column indicate significant differences in MIC and MBC among the various extracts (ANOVA with Tukey’s HSD, *p* < 0.05).

**Table 9:** Minimum inhibitory concentrations (MIC) and minimum bactericidal concentrations (MBC) of the various extracts (A—aqueous; E40 and E60—ethanolic 40% and 60%; and Enz—enzymatic) from *S. triloba*, against pathogens. Mean values (mg/mL) from three repetitions.

	Minimum Inhibitory Concentration (mg/mL)				Minimum Bactericidal Concentration (mg/mL)			
Pathogen	A	E40	E60	Enz	A	E40	E60	Enz
*S. aureus* MRSA/VRSA	12.5 ± 0 ^e^	3.1 ± 0 ^b^	1.6 ± 0 ^b^	6.3 ± 0 ^d^	50 ± 0 ^d^	12.5 ± 0 ^c^	6.3 ± 0 ^d^	25 ± 0 ^f^
MRSA (raw milk)	6.3 ± 0 ^d^	3.1 ± 0 ^b^	1.6 ± 0 ^b^	6.3 ± 0 ^d^	12.7 ± 0.3 ^b^	12.5 ± 0 ^c^	3.1 ± 0 ^c^	25 ± 0 ^f^
MRSA (raw poultry)	12.5 ± 0 ^e^	6.3 ± 0 ^c^	0.8 ± 0 ^a^	3.1 ± 0 ^c^	50 ± 0 ^d^	12.5 ± 0 ^c^	3.1 ± 0 ^c^	12.5 ± 0 ^e^
*S. mutans*	0.8 ± 0 ^a^	6.3 ± 0 ^c^	3.1 ± 0 ^c^	0.8 ± 0 ^a^	6.3 ± 0 ^a^	25 ± 0 ^d^	12.5 ± 0 ^d^	3.1 ± 0 ^c^
*S. salivarius*	3.1 ± 0 ^c^	3.1 ± 0 ^b^	0.8 ± 0 ^a^	6.3 ± 0 ^d^	12.7 ± 0.3 ^b^	6.3 ± 0 ^b^	1.6 ± 0 ^b^	12.5 ± 0
*P. gingivalis*	0.8 ± 0 ^a^	6.3 ± 0 ^c^	6.3 ± 0 ^d^	0.8 ± 0 ^a^	6.3 ± 0 ^a^	25 ± 0 ^d^	12.5 ± 0 ^d^	1.6 ± 0 ^b^
*F. nucleatum*	12.5 ± 0	6.3 ± 0 ^c^	1.6 ± 0 ^b^	1.6 ± 0 ^b^	50 ± 0 ^d^	12.5 ± 0 ^c^	3.1 ± 0 ^c^	1.6 ± 0 ^b^
*P. intermedia*	1.6 ± 0 ^b^	3.1 ± 0 ^b^	0.8 ± 0 ^a^	3.1 ± 0 ^c^	12.7 ± 0.3 ^b^	12.5 ± 0 ^c^	0.8 ± 0 ^a^	6.3 ± 0 ^d^
*P. micra*	1.6 ± 0 ^b^	1.6 ± 0 ^a^	0.8 ± 0 ^a^	0.4 ± 0 ^a^	6.3 ± 0 ^a^	3.1 ± 0 ^a^	0.8 ± 0 ^a^	1.6 ± 0 ^b^
*S. aureus* ATCC 12600 (Ref)	6.3 ± 0 ^d^	1.6 ± 0 ^a^	0.8 ± 0 ^a^	0.7 ± 0.2 ^a^	25 ± 0 ^c^	3.1 ± 0 ^a^	3.1 ± 0 ^c^	0.8 ± 0 ^a^

Values are the mean of three replicates. Different superscript letters in a column indicate significant differences in MIC and MBC among the various extracts (ANOVA with Tukey’s HSD, *p* < 0.05).

**Table 10:** Percentage (%) of hemolysis of human blood type “AB” erythrocytes treated with various *O. vulgare* and *S. triloba* extracts as compared to the positive control.

	Concentration of Each Plant Extract (μg/mL)								
	12.5	25	50	100	150	200	250	500	1000
Oregano A	4.35 ± 1 ^1de^	4.21 ± 0.6 ^1bc^	4.46 ± 0.7 ^1cde^	5.45 ± 0.3^1bc^	7.57 ± 0.3^2c^	8.45 ± 0.1 ^23c^	8.64 ± 0.1 ^23bcd^	9.37 ± 0.2 ^34ab^	10.1 ± 0.5 ^4ab^
Oregano A *	2.74 ± 0.5 ^1bc^	3.81 ± 0.7 ^1bc^	4.54 ± 0.6 ^12de^	6.04 ± 0.7^2bcd^	9.04 ± 0.7^3d^	10.43 ± 0.5 ^34c^	11.34 ± 0.7 ^4cde^	19.9 ± 0.9 ^5c^	21.62 ± 0.4 ^5cd^
Oregano E40	3.55 ± 0.1 ^1cd^	4.1 ± 0.9 ^12bc^	4.17 ± 0.9 ^12cd^	7.03 ± 1.4^12cd^	9.07 ± 0.7^12d^	14.42 ± 3.3 ^23d^	21.51 ± 1.6 ^3f^	34.39 ± 2.6 ^4d^	54.92 ± 9.7 ^5e^
Oregano E60	4.94 ± 0.6 ^1de^	6.07 ± 0.4 ^1d^	6.62 ± 0.5 ^1f^	9.04 ± 1.1 ^1a^	10.68 ± 0.3 ^1e^	22.14 ± 1.2 ^2ce^	29.09 ± 1 ^3g^	31.76 ± 0.5 ^3d^	82.58 ± 5.9 ^4f^
Oregano ENZ	0.55 ± 0.4 ^1a^	1.43 ± 0.5 ^1a^	1.94 ± 0.1 ^1ab^	4.5 ± 0.1 ^12b^	7.24 ± 0.2 ^2c^	8.01 ± 0.5 ^2^	14.38 ± 4.1 ^3e^	29.42 ± 0.2 ^4d^	31.1 ± 0.5 ^4^
Salvia A	0.88 ± 0.2 ^1ab^	1.32 ± 0.1 ^12a^	1.9 ± 0.1 ^23ab^	2.49 ± 0.2 ^3a^	2.89 ± 0.3 ^4a^	4.06 ± 0.4 ^5ab^	4.76 ± 0.6 ^5ab^	6.29 ± 0.3 ^6a^	7.46 ± 0.3 ^7a^
Salvia A*	1.39 ± 0.4 ^1a^	1.61 ± 0.3 ^1a^	3.04 ± 0.7 ^2bc^	4.46 ± 0.3 ^3b^	5.67 ± 0.3 ^4b^	7.1 ± 0.5 ^5bc^	8.23 ± 0.5 ^6abc^	9.04 ± 0.1 ^67a^	9.59 ± 0.1 ^7ab^
Salvia E40	2.6 ± 0.3 ^1bc^	3.33 ± 0.1 ^12b^	3.81 ± 0.2 ^12cd^	4.9 ± 0.4 ^12b^	5.74 ± 0.4 ^123b^	7.24 ± 0.2 ^123bc^	8.6 ± 0.2 ^23bcd^	6.41 ± 5.6 ^3a^	10.76 ± 0.2 ^3ab^
Salvia E60	3.77 ± 0.3 ^1cde^	4.87 ± 0.4 ^12cd^	5.63 ± 0.2 ^2ef^	7.32 ± 0.1 ^34de^	8.12 ± 0.8 ^4cd^	10.06 ± 1 ^5c^	12.99 ± 0.9 ^6e^	14.89 ± 0.4 ^7bc^	19.72 ± 0.8 ^8bc^
Salvia ENZ	0.18 ± 0.3 ^1a^	0.8 ± 0.1 ^12a^	0.91 ± 0.1 ^12a^	1.24 ± 0.1 ^2a^	2.56 ± 0.5 ^3a^	3.11 ± 0.2 ^34a^	3.92 ± 0.3 ^4a^	5.89 ± 0.6 ^5a^	7.98 ± 0.7 ^6a^

A*: A solution of an aqueous extract dissolved in a solvent consisting of equal parts (1:1 ratio) boiled water and 95% methanol. Values are the mean of three replicates. Similar superscript numbers indicate no statistically significant differences in rows and similar superscript letters indicate no statistically significant differences in columns (ANOVA with 95.0% Tukey HSD).

## Data Availability

Data are contained within the article and supplementary materials.

## Supplementary Materials

The following supporting information can be downloaded at: https://www.mdpi.com/article/10.3390/biom14060619/s1, Table S1: Total phenolics and flavonoid content of *O. vulgare* and *S. triloba* plant extracts; Figure S1: Hemolytic activity of the various *O. vulgare* extracts in human blood type O erythrocytes compared to the negative and positive (Triton X-100) controls; Figure S2: Hemolytic activity of the various *S. triloba* extracts in human blood type O erythrocytes compared to the negative and positive (Triton X-100) controls.
