# Supplementary material for: Comprehensive Bio-Screening of Phytochemistry and Biological Capacity of Oregano (Origanum vulgare) and Salvia triloba Extracts against Oral Cariogenic and Food-Origin Pathogenic Bacteria
Source: Biomolecules. 2024 May 24;14(6):619. doi: 10.3390/biom14060619 (PMC11201555; doi:10.3390/biom14060619)
Supplement: Supplementary file 1 [file biomolecules-14-00619-s001.zip › biomolecules-3017483-supplementary.pdf]

Table S1. Total phenolics and flavonoid content of *O. vulgare* and *S. triloba* plant extracts

Plant extracts	Total phenolics (mg gallic acid equivalent/g of dried sample)	Total Flavonoids (mg catechin equivalent per /g of dried sample)
Oregano A	39.69±0.54[b]	8.13±0.82[a]
Oregano A*	45.46±0.8[c]	15.9±0.38[b]
Oregano E40	76.68±3.34[e]	22.56±0.48[c]
Oregano E60	88.44±0.6[f]	30.74±0.98[d]
Oregano ENZ	121.4±0.6[g]	48.83±1.17[f]
Salvia A	91.99±1.08[f]	55.31±0.35[g]
Salvia A*	79±0.8[e]	37.58±1.15[e]
Salvia E40	50.76±0.6[d]	37.56±0.47[e]
Salvia E60	22.44±0.6[a]	39.67±0.44[e]
Salvia ENZ	90.42±0.5[f]	64.75±0.65[h]

A*: A solution of an aqueous extract dissolved in a solvent consisting of equal parts (1:1 ratio) boiled water and 95% methanol. Values are the mean of three replicates (TPC). Different letters in columns denote statistical differences between the total phenolics or total flavonoids among the various extracts in each plant (Kruskal-Wallis, $p<0.05$ with Tukey's HSD).

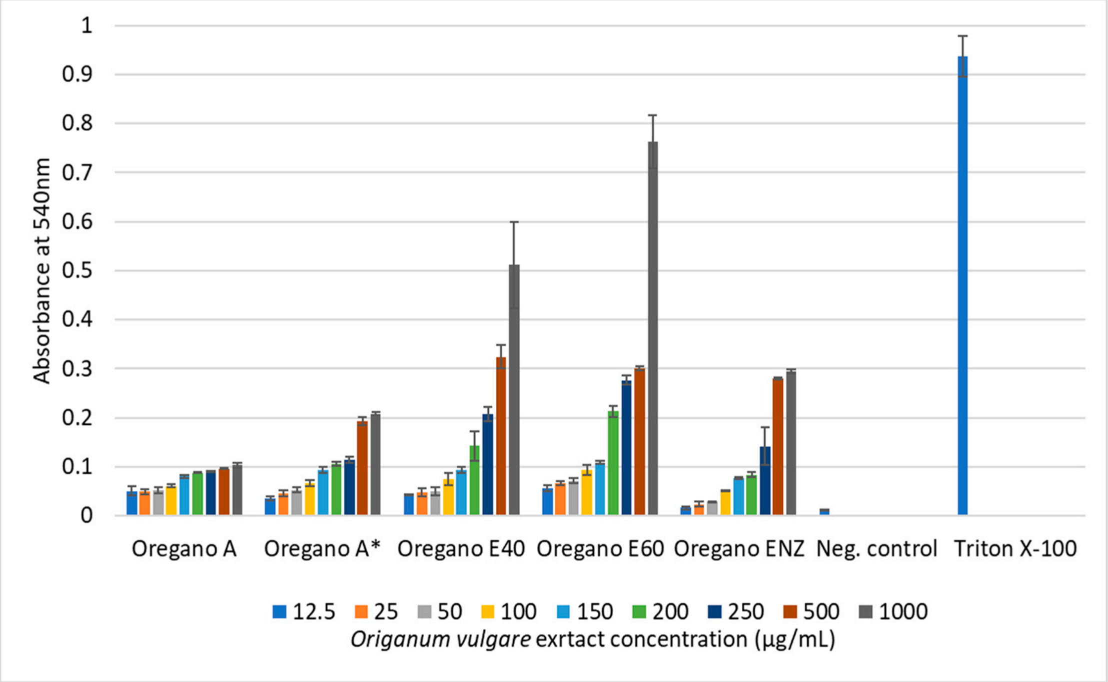

Figure S1. Hemolytic activity of the various *O. vulgare* extracts in human blood type O erythrocytes compared to the negative and positive (Triton X-100) controls

A*: A solution of an aqueous extract dissolved in a solvent consisting of equal parts (1:1 ratio) boiled water and 95% methanol.

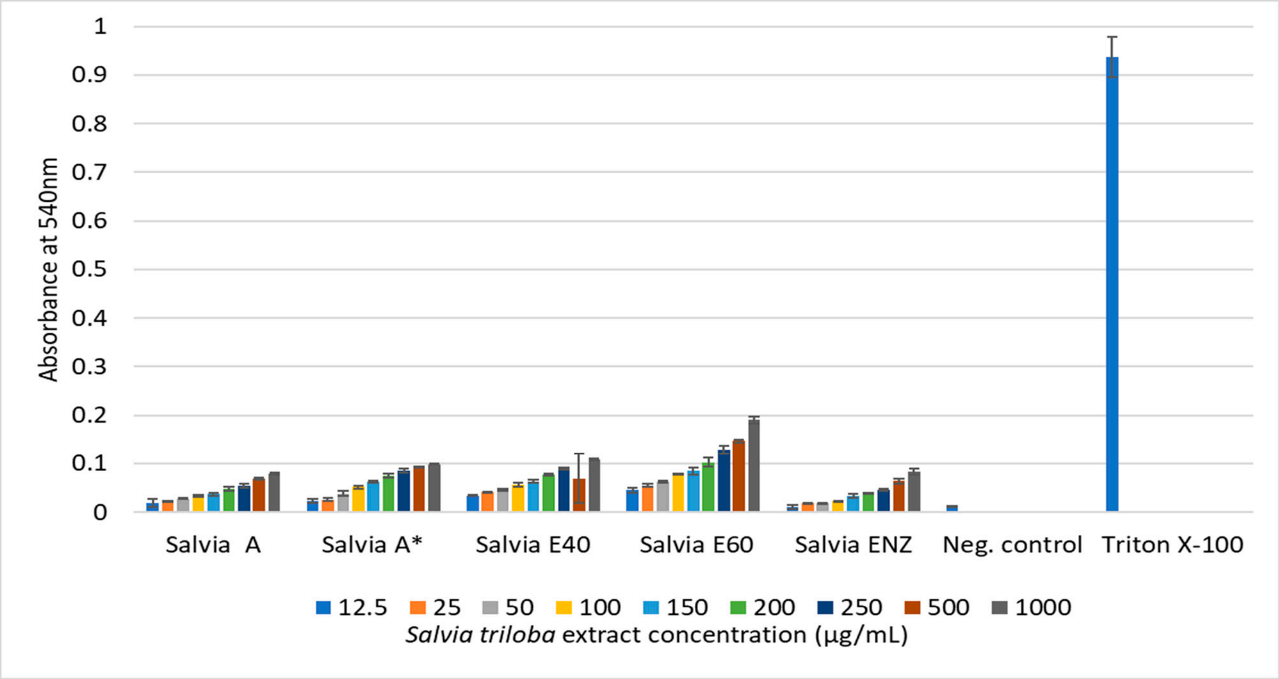

Figure S2. Hemolytic activity of the various *S. triloba* extracts in human blood type O erythrocytes compared to the negative and positive (Triton X-100) controls.

A*: A solution of an aqueous extract dissolved in a solvent consisting of equal parts (1:1 ratio) boiled water and 95% methanol.
